# Solubilization, purification, and characterization of the hexameric form of phosphatidylserine synthase from *Candida albicans*

**DOI:** 10.1016/j.jbc.2023.104756

**Published:** 2023-04-26

**Authors:** Yue Zhou, Jawhar H. Syed, Dmitry A. Semchonok, Edward Wright, Fotis L. Kyrilis, Farzad Hamdi, Panagiotis L. Kastritis, Barry D. Bruce, Todd B. Reynolds

**Affiliations:** 1 Department of Microbiology, University of Tennessee at Knoxville, Knoxville, Tennessee, USA; 2 Department of Biochemistry Cellular and Molecular Biology, University of Tennessee at Knoxville, Knoxville, Tennessee, USA; 3 Interdisciplinary Research Center HALOmem, Institute of Biochemistry and Biotechnology, Martin Luther University Halle-Wittenberg, Halle, Germany

**Keywords:** membrane enzyme, membrane lipid, electron microscopy (EM), enzyme purification, oligomer, fungi

## Abstract

Phosphatidylserine (PS) synthase from *Candida albicans*, encoded by the *CHO1* gene, has been identified as a potential drug target for new antifungals against systemic candidiasis. Rational drug design or small molecule screening are effective ways to identify specific inhibitors of Cho1, but both will be facilitated by protein purification. Due to the transmembrane nature of Cho1, methods were needed to solubilize and purify the native form of Cho1. Here, we used six non-ionic detergents and three styrene maleic acids (SMAs) to solubilize an HA-tagged Cho1 protein from the total microsomal fractions. Blue native PAGE and immunoblot analysis revealed a single band corresponding to Cho1 in all detergent-solubilized fractions, while two bands were present in the SMA2000-solubilized fraction. Our enzymatic assay suggests that digitonin- or DDM-solubilized enzyme has the most PS synthase activity. Pull-downs of HA-tagged Cho1 from the digitonin-solubilized fraction reveal an apparent MW of Cho1 consistent with a hexamer. Furthermore, negative-staining electron microscopy analysis and AlphaFold2 structure prediction modeling suggest the hexamer is composed of a trimer of dimers. We purified Cho1 protein to near-homogeneity as a hexamer using affinity chromatography and TEV protease treatment, and optimized Cho1 enzyme activity for manganese and detergent concentrations, temperature (24 °C), and pH (8.0). The purified Cho1 has a *K*_m_ for its substrate CDP-diacylglycerol of 72.20 μM with a *V*_max_ of 0.079 nmol/(μg∗min) while exhibiting a sigmoidal kinetic curve for its other substrate serine, indicating cooperative binding. Purified hexameric Cho1 can potentially be used in downstream structure determination and small drug screening.

The incidence of human systemic fungal infections has increased dramatically over the past 30 years, and this is partly due to an increase in the number of immunocompromised patients (1, 2, 3). *Candida* species are the leading cause of fungal infections in humans. Among *Candida* spp., *Candida albicans* is the most commonly isolated one, and it is capable of causing mucosal, cutaneous, and bloodstream infections (*i.e.*, invasive mycoses) (1, 4, 5). In all cases, successful management of patients with systemic *Candida* infections requires antifungal therapy. Currently, three classes of antifungals are used against systemic *Candida* infection: azoles, echinocandins, and polyenes. Unfortunately, all of these antifungals have limitations, including rising drug resistance to azoles and echinocandins and toxicity of amphotericin B to patients (6, 7, 8, 9, 10). Therefore, new antifungal drug development is of utmost importance.

The phosphatidylserine (PS) synthase (gene name: *CHO1*) in *C. albicans* has been identified as a potential drug target due to its importance in fungal virulence, conservation among major fungal pathogens, and absence in humans (11, 12, 13). Rational drug design is one way to identify inhibitors of *C. albicans* Cho1 protein, and ligand or structure-based design approaches can be used (14). Given the limited known Cho1 ligands, a structure-based design is more approachable for Cho1, which requires the identification of the substrate-binding pockets. Cho1 has two substrates, cytidyldiphosphate-diacylglycerol (CDP-DAG) and L-serine, from which it catalyzes the formation of the important phospholipid, phosphatidylserine. This catalytic activity has been observed in the mitochondria and endoplasmic reticulum (15, 16, 17). PS is then trafficked to other cellular compartments, such as the plasma membrane. Due to its CDP-DAG binding site, Cho1 belongs to the CDP-alcohol phosphatidyltransferase (CDP-AP) protein family, which has a highly conserved CDP-alcohol phosphotransferase (CAPT) motif, D-(X)_2_-D-G-(X)_2_-A-R-(X)_2_-N-(X)_5_-G-(X)_2_-L-D-(X)_3_-D (18). The CAPT motif is utilized by CDP-AP enzymes to bind CDP-linked molecules and catalyzes the formation of a phosphodiester bond between the CDP-linked molecule and a second small alcohol (13, 19, 20, 21, 22). Several residues within the CAPT motif of *C. albicans* Cho1 have been previously characterized and shown to be essential for function (18). The binding pocket for serine, however, is not conserved among CDP-APs since serine is more specific to Cho1. Recently, the serine-binding pocket of the PS synthase in the archaea *Methanocaldococcus jannaschii* has been identified from its solved structure (23), and some critical serine-binding residues of *C. albicans* Cho1 protein have been identified by alanine scanning mutagenesis (18), providing new details of the serine-binding site in Cho1. However, there are a number of substantial differences between the *M. jannaschii* PS synthase and *C. albicans* Cho1. The Cho1 enzyme is predicted to have only six transmembrane domains, whereas the *M. jannaschii* PS synthase has eight, and there are several key residues that we have identified in Cho1 that do not have a clear role in the *M. jannaschii* PS synthase (18, 23). Thus, it is important to generate a specific structure for the fungal enzyme to further rational drug design approaches.

In addition, there have been only six CDP-AP enzymes within the protein family with solved structures (22, 23, 24, 25, 26, 27) and all are from prokaryotes. Moreover, with the exception of *M. jannaschii* PS synthase, all of the others have six transmembrane domains. A homology model of *C. albicans* Cho1 was previously reported, and it was based on the phosphatidylinositol phosphate synthase from *Renibacterium salmoninarum* (18), which also has six transmembrane domains. The *M. jannaschii* PS synthase structure had also not yet been published at that time. However, due to the low amino acid sequence similarity between *C. albicans* Cho1 and the six existing CDP-APs with known structures, an atomic structure of Cho1 is needed for an unbiased and thorough structure-based design, which requires a relatively homogenous purified *C. albicans* Cho1 enzyme. AlphaFold2 is a powerful tool for structure prediction, but it has limitations such as low efficacy in predicting enzyme binding sites accurately for drug screening (28, 29, 30, 31).

Small molecule screening is another way to identify inhibitors to Cho1. A compound, SB-224289, has been discovered from a previous whole-cell-based screening. Still, it was found to act only on Cho1-related physiological pathways instead of the enzyme itself, preventing further characterization and optimization (32). The major limitation of cell-based drug screening is that it typically fails to find inhibitors of enzymes with known molecular targets and mechanisms (14). In this regard, a target-based screen is more favorable, as it overcomes the limitations that can occur due to cellular entry. These issues can be resolved later through medicinal chemistry approaches. Thus, a successful purification scheme for functional *C. albicans* Cho1 enzyme is necessary.

PS synthase was first identified and purified to homogeneity in *Escherichia coli* (33, 34, 35). In contrast with the membrane-bound characteristics of Cho1 in yeasts, the *E. coli* PS synthase is associated with ribosomes (36). The first fungal Cho1 homolog, from *Saccharomyces cerevisiae*, was identified in 1980 (37, 38), and characterization of the *S. cerevisiae* Cho1 enzyme included an understanding of its regulation (39, 40, 41) and identification of its localization (15, 16). Solubilization and purification were also performed on *S. cerevisiae* Cho1, followed by the characterization of its enzyme kinetics (42, 43, 44). For *C. albicans* Cho1, the structural gene and function were characterized in 2010 (11). Later, the Michaelis-Menten kinetics of the *C. albicans* Cho1 protein were biochemically determined using crude membranes from the wild-type and Cho1-overexpressor strains, which yielded a millimolar-scale *K*_m_ for serine and micromolar-scale *K*_m_ for CDP-DAG (18, 45).

In this study, we aimed to solubilize and purify functional Cho1 protein from its native host, *C. albicans*, and characterize its basic biochemical properties. Previously, Triton X-100 was used to solubilize *S. cerevisiae* Cho1, and subsequent purification was achieved using a lengthy process of CDP-diacylglycerol-Sepharose affinity chromatography followed by anion-exchange DE-53 chromatography, due to the lack of knowledge of the structural gene (43, 44). Even if some insights can be gained from the *S. cerevisiae* Cho1 solubilization/purification, the detergent has to be carefully selected for *C. albicans* Cho1 because the phospholipid compositions of these two species are quite different, thus the optimal solubilization detergent and the conditions are not interchangeable (46, 47, 48). Moreover, since the original purification of *S. cerevisiae* Cho1 in the early 1980s, a number of improved detergents and solubilizing reagents have been created. For example, styrene maleic acid (SMA) copolymers have gained popularity in recent years for membrane protein solubilization as they maintain the original and stable lipid environment, and thus can be a substitute for detergents (47, 48, 49, 50). In this work, several popular detergents and SMAs were screened and optimized to solubilize Cho1 from the crude membrane with high activity and homogeneity. The assessment of the solubilized *C. albicans* Cho1 by blue-native (BN)-PAGE and several complementary approaches indicated that *C. albicans* Cho1, unlike *S. cerevisiae* Cho1 or any other CDP-AP, is a hexamer. The purified *C. albicans* Cho1 can be used for downstream applications such as small molecule screening or structure determination.

## Results

### Solubilization of *C. albicans* Cho1 with different detergents and SMA copolymers

**Figure 1:**
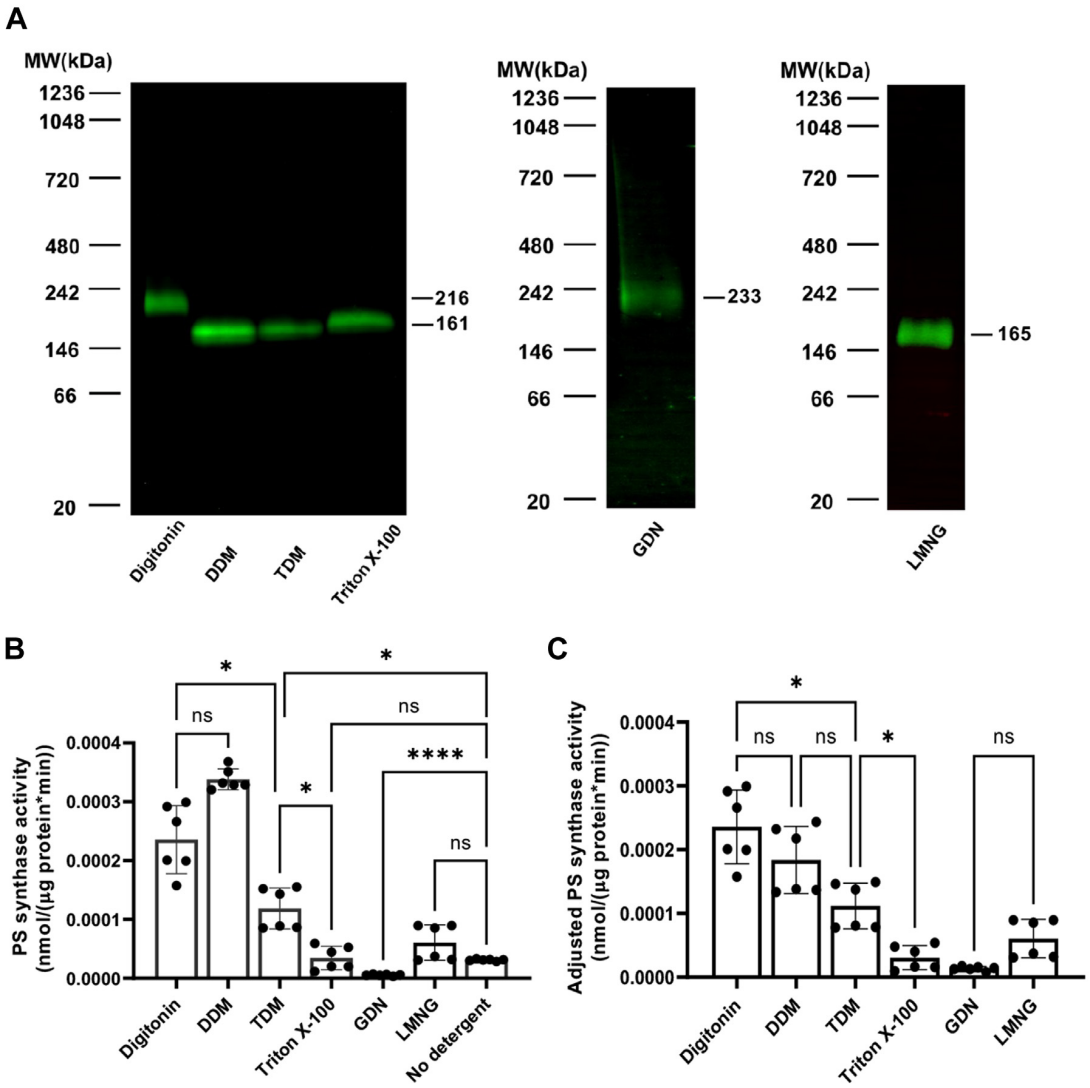
**Solubilization of HAx3-tagged Cho1 using six popular non-ionic detergents.***A*, crude membrane was collected from the *cho1*ΔΔ *P*_*ENO1*_*-CHO1-HAx3* (HA1) strain. Digitonin, DDM, TDM, Triton X-100, GDN, or LMNG were used to solubilize Cho1 at a final concentration of 1.5%. The solubilized Cho1-HAx3 protein was detected by Western blotting of the BN-PAGE. For each solubilized fraction, (*B*) the PS synthase activity (nmol/(μg protein∗min)) and (*C*) adjusted PS synthase activity (nmol/(μg protein∗min)) were measured by an *in vitro* PS synthase assay. Crude membrane protein with no detergent was used as a control. Statistics were conducted using one-way ANOVA and Dunnett's T3 multiple comparisons test (ns = not significant, *p* > 0.05; ∗0.05 > *p* > 0.01; ∗∗∗∗*p* < 0.0001). The activities were measured in duplicate with a total of six biological replicates as indicated. The bars represent the mean, and the error bars are ± standard deviation (S.D.) values.

Since Cho1 is a transmembrane protein, solubilization was conducted before protein purification. The *C. albicans* strain HA1 is a derivative of SC5314 and has a C-terminally HAx3-tagged Cho1 protein under the constitutively active *ENO1* promoter (18). This strain was used to express the Cho1 protein for solubilization. To determine the optimal detergent for solubilization, total crude membranes were collected and solubilized using the detergents digitonin, n-Dodecyl-β-D-Maltopyranoside (DDM), n-Tetradecyl-β-D-Maltopyranoside (TDM), Triton X-100, the digitonin substitute (Glyco-Diosgenin, GDN), or Lauryl Maltose Neopentyl Glycol (LMNG), each at a final concentration of 1.5%. To check if the Cho1 protein was successfully solubilized from the membrane, as well as to measure the molecular weight (MW) of the native protein complex, the resulting solubilized detergent fractions were assessed by BN-PAGE followed by Western blotting against the HA epitope to identify the Cho1 complex. All six detergents led to the formation of a single band representing the HA-tagged Cho1 (Fig. 1*A*), confirming that these detergents could successfully solubilize Cho1 native membranes. However, the estimated MW of digitonin and GDN solubilized Cho1 is higher than the others, indicating a difference in (i) the conformation of the solubilized Cho1 protein, (ii) the composition of residual bound phospholipids in the Cho1-micelle particle, or (iii) the extent to which excess lipid interferes with protein migration in the protein-detergent micelles. These factors have been previously shown to affect protein migration during BN-PAGE (49).

The PS synthase activity of each detergent-solubilized Cho1 fraction was measured and compared to the original crude membrane prep (no detergent fraction) (Fig. 1*B*). The PS synthase activity was calculated based on the incorporation of L-[^3^H]-serine into the lipid phase following normalization to the protein quantity, as described previously (18, 45). Digitonin-, DDM-, and TDM-solubilized fractions showed significantly increased PS synthase activity compared to the crude membrane (Fig. 1*B*). In contrast, Triton X-100- and LMNG-solubilized fractions have similar activity to the unextracted membranes, and the GDN-solubilized fraction had diminished activity. The DDM-solubilized fraction has the highest PS synthase activity and is followed by the digitonin fraction. The higher PS synthase activity in these two detergent fractions can be explained by (i) a higher solubilization rate (thus, more Cho1 protein molecules are present in the reaction), (ii) a solubilized conformation with higher enzyme activity, or (iii) both.

To help explain these observations, the adjusted PS synthase activity was calculated using PS synthase activity adjusted to the Cho1 protein level reflected on the immunoblots. This adjusted PS synthase activity could better represent the enzymatic activity of Cho1 in the solubilized fractions since the activity was further normalized to the Cho1 protein level in the solubilized fractions. In Figure 1*C*, the digitonin- and DDM-solubilized fractions showed similar levels of adjusted PS synthase activity as opposed to the slightly increased PS synthase activity in the DDM fraction, indicating that the high activity of PS synthase activity in the DDM fraction is due to a higher solubilization rate rather than a higher enzymatic activity. On the contrary, the lower PS synthase activities of Triton X-100, GDN-, and LMNG-solubilized fractions are due to the lower enzymatic activity of solubilized Cho1 in these detergents.

**Figure 2:**
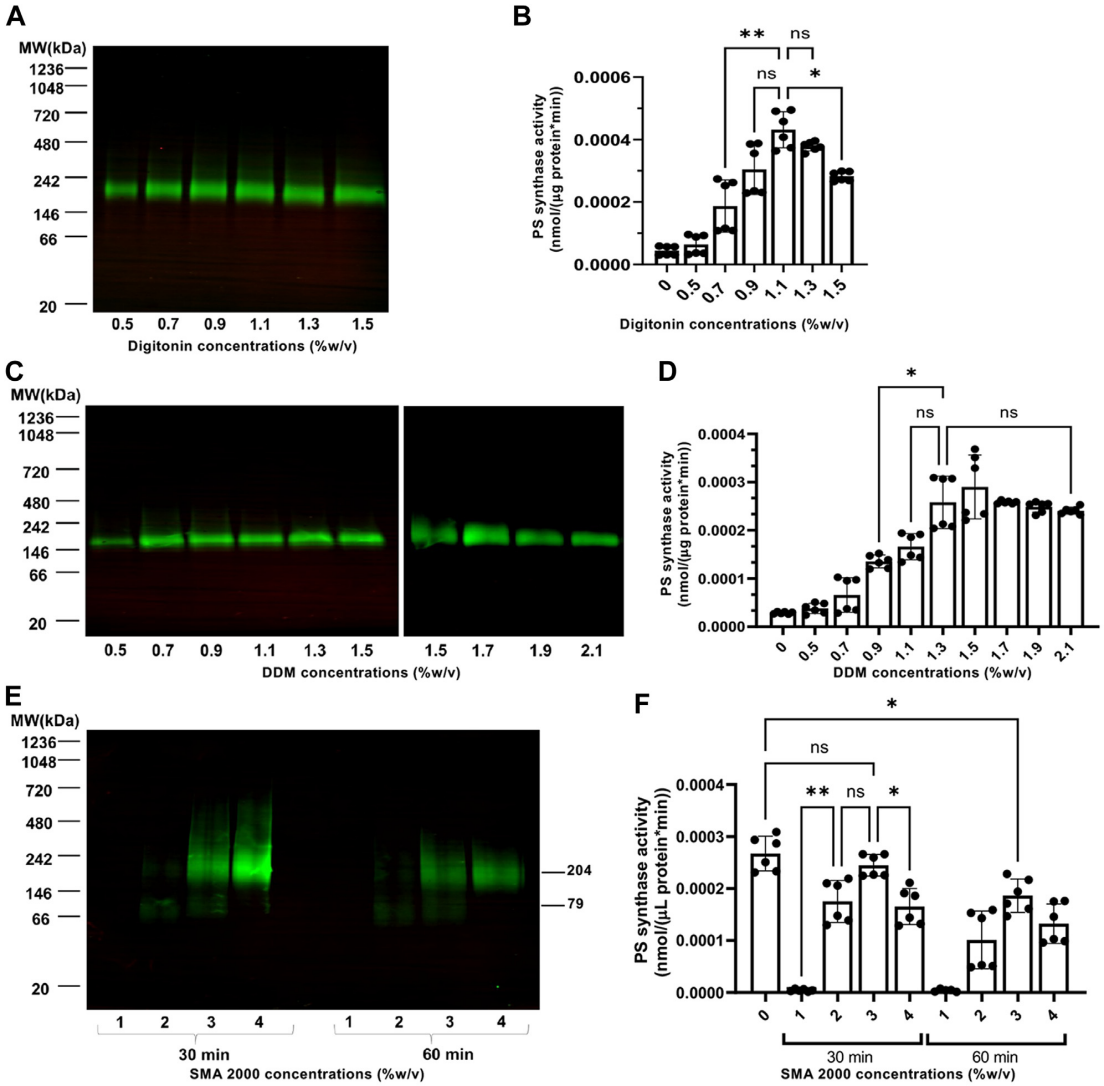
**Optimization of digitonin, DDM, and SMA 2000 for solubilization and activity.***A* and *C*, HAx3-tagged Cho1 protein was solubilized in different (*A*) digitonin concentrations (0.5%, 0.7%, 0.9%, 1.1%, 1.3% and 1.5%) or (*C*) DDM concentrations (0.5%, 0.7%, 0.9%, 1.1%, 1.3%, 1.5%, 1.7%, 1.9% and 2.1%), and Cho1 levels for the different fractions were measured by Western blotting following BN-PAGE. *B* and *D*, fractions were also tested for PS synthase activity (nmol/(μg protein∗min)). Digitonin-solubilized fractions are shown in (*B*) and DDM-solubilized fractions are in (*D*). *E*, SMA2000-solubilized HAx3-tagged Cho1 protein, at different times (30 or 60 min) and SMA concentrations (1%, 2%, 3% and 4%), was detected by Western blot. *F*, the PS synthase activities of SMA 2000-solubilized fractions were measured and are presented as nmol/(μl protein∗min). Crude membrane protein with no detergent/SMA2000 was used as a control. Statistics were conducted using one-way ANOVA and Dunnett's T3 multiple comparisons test (ns = not significant, *p* > 0.05; ∗0.05 > *p* > 0.01, ∗∗0.01 > *p* > 0.001). The activities were measured in duplicate with a total of six biological replicates as indicated. The bars represent the mean and the error bars are ± S.D. values.

Based on these results, both digitonin and DDM were selected for further optimization. For this optimization, different concentrations of digitonin and DDM were used to solubilize the Cho1 protein. The resulting solubilized fractions were subjected to conformational (BN-PAGE) and activity checks. For digitonin, all six concentrations, 0.5%, 0.7%, 0.9%, 1.1%, 1.3%, and 1.5%, were able to solubilize Cho1 and 1.1% was determined to be the optimal concentration as its PS synthase activity is highest (Fig. 2, *A* and *B*). DDM's concentration was increased from 0.5% to 2.1% in 0.2% increments, and PS synthase activity remained unchanged at ≥1.3% DDM (Fig. 2, *C* and *D*).

SMA copolymers have gained popularity in the membrane protein field as they are a substitution for detergents to produce an SMA Lipid Particle (SMALP) that maintains a membrane protein's original and stable lipid environment (50, 51, 52, 53). SMALPs are compatible with many biophysical approaches (50, 51, 54). Initially, SMA1000, 2000, and 3000 were used to solubilize Cho1 at concentrations of 2%, 3%, and 4% at 37 °C, but only SMA2000 was able to solubilize Cho1 from the membrane (Fig. S1). SMA2000 was further refined, and a range of 1% to 4% SMA2000 was used to optimize the solubilization conditions with either 30- or 60-min incubations (Fig. 2, *E* and *F*). Two bands (204 kDa and 79 kDa) of Cho1 were found at concentrations of 2% to 4% even if the lower band has much less protein, indicating two distinct protein species. Moreover, solubilization at 30 min had a higher yield than at 60 min, possibly due to protein degradation or dissociation of the SMALP complex. SMA2000 at 4% gave the highest protein yield after a 30 min solubilization (Fig. 2*E*). In contrast, enzyme assays revealed that the 3% SMA2000 solubilized fraction had the highest PS synthase activity (Fig. 2*F*). Since the protein concentrations in the SMA-solubilized fractions were not measurable due to interference of the SMA copolymers with protein concentration assays (data not shown), PS synthase activities for these fractions were normalized by the volume of the reaction (nmol/(μl protein∗min)). One explanation for the inconsistency between Cho1 band intensity and activity at 4% is that excessive SMA2000 in this fraction may interfere with the activity, which offsets the high protein amount. The SMA2000 bands were much more diffuse than those in the detergent fractions. Since detergent fractions gave the greatest increases in activity and the clearest bands, further purification schemes were pursued with the detergents digitonin and DDM.

### *C. albicans* Cho1 appears as a hexamer

**Figure 3:**
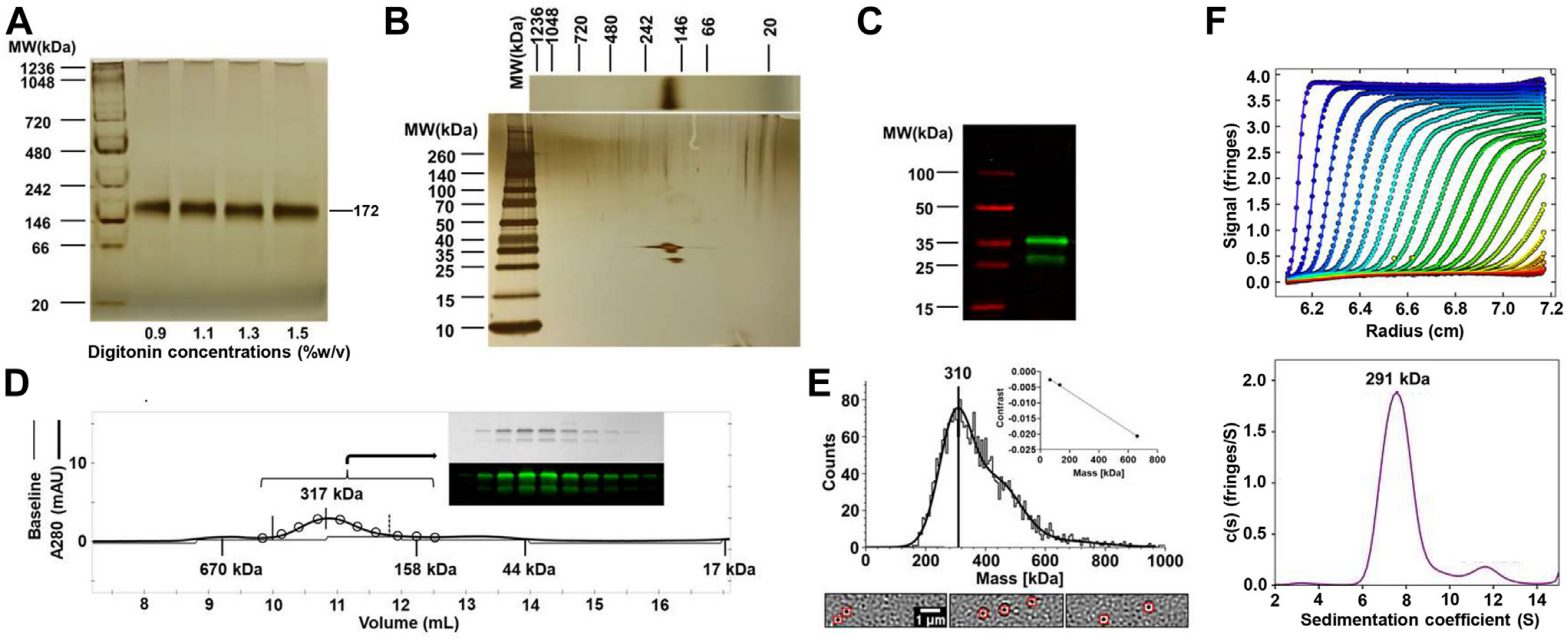
**The solubilized native Cho1 complex appears to be a hexamer.***A*, BN-PAGE of the pulled-down HAx3-tagged Cho1 protein from the solubilized fractions of 0.9%, 1.1%, 1.3%, 1.5% digitonin. *B*, the second dimensional SDS-PAGE of the BN-PAGE gel strip from 1.1% digitonin. These BN-PAGE gels were stained with a Pierce silver stain kit, and MWs of different bands (kDa) were estimated based on the protein ladder as indicated. *C*, SDS-PAGE and Western blot of the pulled-down HAx3-tagged Cho1 protein showed three bands at 36 kDa, 29 kDa and 27 kDa, representing the bands from (*B*). *D*, size exclusion chromatography of pulled-down HAx3-tagged Cho1 protein. The estimated molecular weights of Cho1 and different protein markers are indicated. The protein profile was assessed by the eluted fractions (*circles*) on silver-stained SDS-PAGE (inset figure, *top*) or Western blotting with anti-HA antibody (inset figure, *bottom*). *E*, histogram of trajectory counts detected in a mass photometry movie (n = 1 movie, 1 min) of pulled-down HAx3-tagged Cho1 protein. Contrast–mass calibration curve of the measurement is shown in the inset (BSA, 66 kDa and 132 kDa; Thyroglobulin, 660 kDa). Three frames with Cho1-digitonin-micelles (highlighted in *red squares*) are shown under the histogram. *F*, sedimentation velocity analytical ultracentrifugation (SV-AUC) analysis of the oligomeric state of Cho1. SV-AUC interference profile for Cho1 sedimenting at 50,000 rpm at 20 °C. The raw data (*circles*) and best fit using the continuous c(s) distribution model (*lines*) in SEDFIT are shown on the *top*. Every fourth scan is shown for clarity. The c(s) distribution plot for the data in the *top panel* is shown on the *bottom*. The peak at 7.7S accounts for over 85% of the total signal. This peak corresponds to an MW of 291 kDa as calculated in SEDFIT using the buffer density and viscosity, protein partial specific volume, and best fit frictional coefficient.

Next, to assess the feasibility of purifying Cho1 from solubilized fractions, a small-scale pull-down was performed using anti-HA beads, and BN-PAGE measured pulled-down proteins from digitonin-solubilized membranes. As shown in Figure 3*A*, a single 172-kDa native complex containing Cho1 was found in the 0.9%, 1.1%, 1.3%, and 1.5% digitonin fractions, indicating high purity. To check the protein composition of this native complex, a second-dimensional SDS-PAGE was performed on the gel strip of the 1.1% digitonin fraction from the BN-PAGE (Fig. 3*B*). This second-dimensional SDS-PAGE, which should break up complexes into individual components, revealed several bands. To determine whether these bands represent Cho1, the same pulled-down sample was subjected to Western blotting against the HA tag (Fig. 3*C*). It revealed one upper band (36 kDa) and two lower bands (29 and 27 kDa), corresponding to the bands on the second dimensional SDS-PAGE based on MW, contained the HA tag. This result indicates that the 172-kDa native complex corresponds to Cho1, suggesting this native complex is made of only Cho1 protein instead of interactions with other proteins. The multiple bands of Cho1 on the SDS-PAGE have been reported previously, and it is suggested that the lower bands are the proteolytic product of the full-length upper band (16, 18, 55). Interestingly, based on the MW of the Cho1 complex (172 kDa) and monomeric HA-tagged Cho1 (27, 29, 36 kDa), the native complex is likely to be a hexamer. In order to measure the molecular mass of the native Cho1 protein and test if Cho1 forms a hexamer by complementary approaches, the pull-down Cho1 protein was analyzed by size-exclusion chromatography (Fig. 3*D*) and mass photometry (Fig. 3*E*), both of which suggested an ∼310 kDa form. Moreover, analytical ultracentrifugation also indicated the MW of the pull-downed Cho1 protein to be 291 kDa (Fig. 3*F*). Unlike BN-PAGE which converts membrane proteins into water-soluble proteins upon binding G250 dye (56), these approaches examine the molecular mass of the native Cho1-digitonin-micelle particle. Since it is hard to determine how many digitonin monomers are associated with Cho1, the mass of an empty digitonin micelle (124 kDa, (57)) was subtracted from the Cho1-digitonin-micelle particle, and the estimated MW of the Cho1 protein alone from these independent approaches is close to ∼180 kDa, as we saw in the native gels, again supporting a hexamer.

For DDM-solubilized fractions, the anti-HA pull-downs in the 0.9%, 1.1%, 1.3%, and 1.5% solubilized particles were subjected to BN-PAGE followed by a second-dimensional SDS-PAGE as described above (Fig. S2). Even though the 168-kDa complex is present at the highest abundance, multiple bands of other molecular weights are also observed on the BN-PAGE. Given that the molecular weight of monomeric Cho1 ranges from 27 to 36 kDa, the various bands are likely to correspond to different oligomeric states of Cho1 because the differences among them are close to the multiples of ∼30 kDa, with the hexamer form (168 kDa) being the most abundant. This is also supported by the second dimensional SDS-PAGE, which only shows bands corresponding to Cho1 in the native complexes. However, since different oligomers exist in the DDM-solubilized fraction, digitonin was chosen as the detergent for the solubilization and subsequent purification as it gives a more consistent oligomeric state.

### Affinity tag construction and protein purification of *C. albicans* Cho1

**Figure 4:**
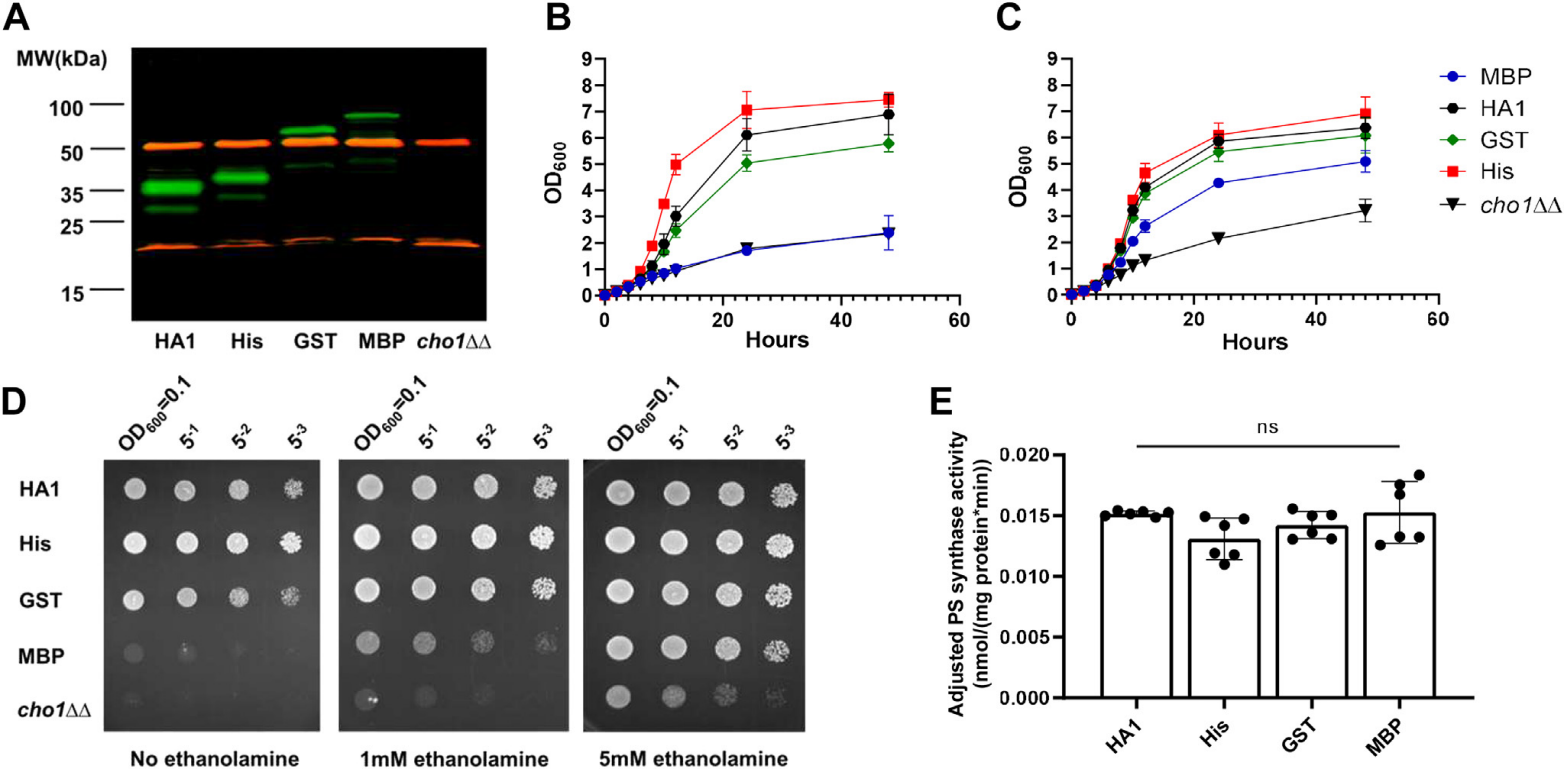
**His-, GST- and MBP-tagged Cho1 construct express active enzyme.***A*, membranes containing Cho1 protein were extracted from the HA1, *cho1*ΔΔ *P*_*ENO1*_*-CHO1-ENLYFQG-HAx3-Hisx8* (Hisx8-tagged Cho1), *cho1*ΔΔ *P*_*ENO1*_*-CHO1-ENLYFQG-HAx3-GST* (GST-tagged Cho1), *cho1*ΔΔ *P*_*ENO1*_*-CHO1-ENLYFQG-HAx3-MBP* (MBP-tagged Cho1), and *cho1ΔΔ* negative control strains, fractionated by SDS-PAGE and Western blotted with anti-HA (*green*) and anti-tubulin (*red*) antibodies. Different bands corresponding to Cho1 appear at their expected MW. *B–D*, *in vivo* activities of Cho1 with different tags were assessed *via* growth curves in the absence (*B*) and presence (*C*) of 1 mM ethanolamine, and (*D*) corresponding spot dilution assays on agar plates. *E*, adjusted PS synthase activities (nmol/(mg protein∗min)) of the crude membrane fractions from different Cho1 strains were measured. Statistics were conducted using one-way ANOVA and Dunnett's T3 multiple comparisons test (ns = not significant, *p* > 0.05). The activities were measured in duplicate with a total of six biological replicates as indicated. The bars represent the mean and the error bars are ± SD values.

To achieve a large-scale purification, codon-optimized glutathione S-transferase (GST), maltose-binding protein (MBP), and octa-histidine (Hisx8) tags were C-terminally attached to the existing HAx3 tag of Cho1. A Tobacco Etch Virus (TEV) protease recognition site, ENLYFQG, was inserted between the Cho1 protein and the HAx3 tag to permit the removal of the HA epitope & C-terminal affinity tags after purification. All three versions of the tagged Cho1 protein were expressed from the constitutively active *ENO1* promoter in *C. albicans*, and immunoblotting confirmed their expression (Fig. 4*A*). To determine the best tag for purification, the enzyme activities of these three different affinity-tagged Cho1 proteins were measured by both *in vivo* and *in vitro* assays.

Growth in conditions that require PS synthesis was used as a proxy for *in vivo* activity. Loss of the *CHO1* gene leads to poor growth in general because Cho1 forms PS, which is the first phospholipid in the *de novo* pathway, and is a substrate for synthesizing the essential phospholipid, phosphatidylethanolamine (PE). The *cho1*ΔΔ mutant exhibits perturbed growth in general, but especially in minimal media, which lacks ethanolamine (11, 18). Cell growth can be partially restored by supplementing the media with exogenous ethanolamine, which is consumed in the Kennedy salvage pathway to make PE. Hence, *in vivo* Cho1 activity correlates with cell growth in the minimal media lacking ethanolamine. Therefore, growth curves and spot dilution assays in minimal media were used to measure the growth of strains bearing the three versions of Cho1 (Fig. 4, *B*–*D*) to determine how well each functioned in providing the needed phospholipids. A strain lacking Cho1 (*cho1*ΔΔ) served as a negative control, while the HA1 strain was used as a positive control. Cells with the Cho1-GST tag grew similarly to HA1 in all conditions, while Cho1-MBP did not grow similarly to HA1 and was closer to the *cho1*ΔΔ mutant. This suggests that the MBP tag interferes with the Cho1 function in the cell, unlike the GST tag. In contrast, the Hisx8 tagged Cho1 could complement the growth of the *cho1ΔΔ* mutant and unexpectedly even caused the strain to grow slightly faster than the HA1 strain. This was more evident during growth in minimal media without ethanolamine (Fig. 4, *B*–*D*), suggesting that the Hisx8 tag may improve the activity of Cho1 compared to the HA tag alone *in vivo*.

To measure the *in vitro* activity of these three new Cho1 constructs, the PS synthase activity assay was performed. Instead of the solubilized fraction, crude membranes were used in the reaction as described in Experimental Procedures. The adjusted PS synthase activity (nmol/(mg protein∗min)) was then calculated from the PS synthase activity normalized to Cho1 expression *via* Western blotting, which reflects the intrinsic enzymatic activity of Cho1 with different tags. Contrary to the *in vivo* growth assay results, there is no significant difference in the adjusted PS synthase activities among these different versions of Cho1 (Fig. 4*E*). The full explanation for why there was not a clear correlation between growth and *in vitro* activity for the different Cho1 constructs is unknown. However, the low *in vivo* activity of MBP-tagged Cho1 protein could be due to decreased expression, as supported by the Western blotting results (Fig. 4*A*). The reasons for the difference between Cho1-His and Cho1-GST are unknown, but the better growth of the Cho1-His strain suggested it would be better for large-scale purification.

**Figure 5:**
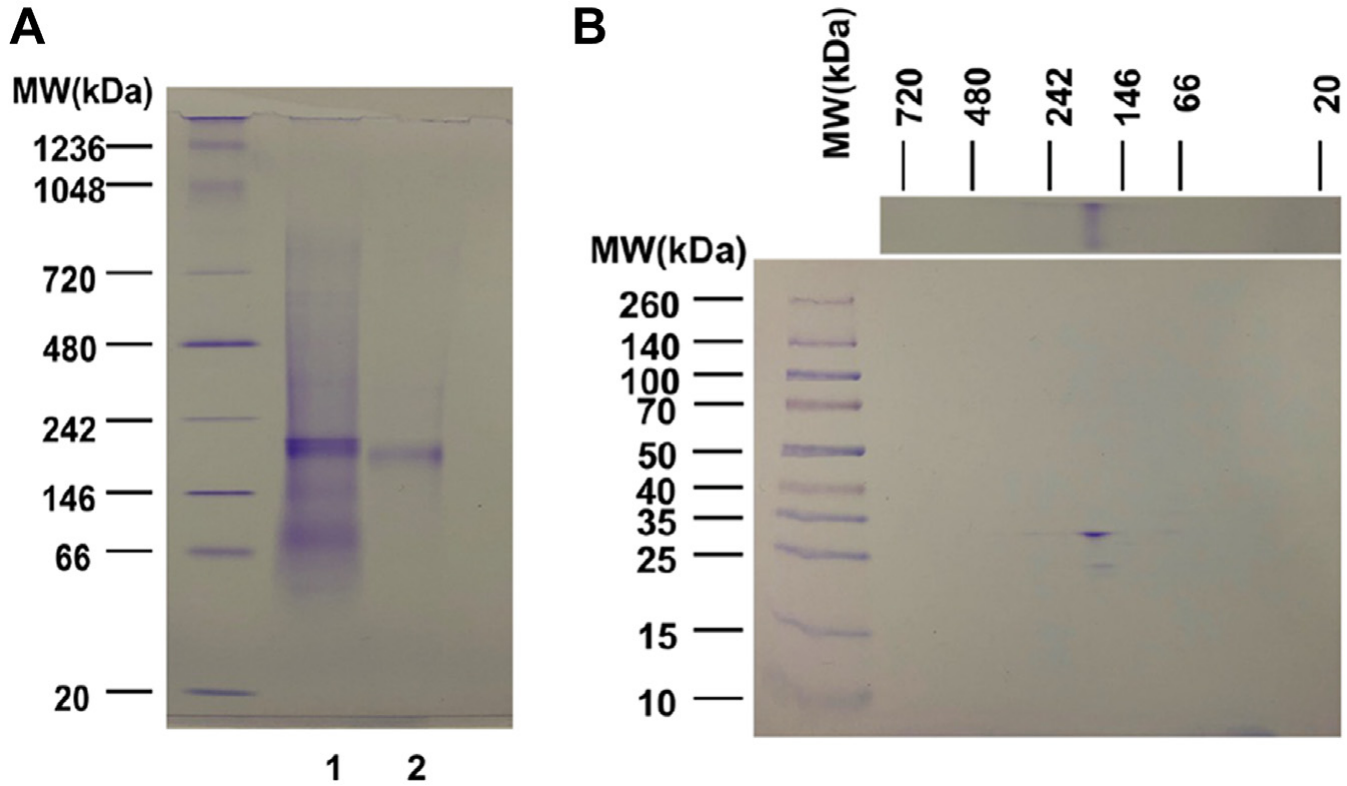
**Tag-free hexameric Cho1 was purified to homogeneity using affinity chromatography and AcTEV treatment.***A*, BN-PAGE gel of the purified hexameric Hisx8-tagged Cho1 protein prior to tag removal (lane 1), and after AcTEV treatment and second purification for the tag-free version (lane 2). *B*, second dimensional SDS-PAGE of lane two from the BN-PAGE in (*A*) shows bands corresponding to Cho1 based on MW. All gels were stained with Coomassie blue R250 dye.

In addition, Hisx8-tagged Cho1 was optimal due to its more specific binding/elution conditions, and a large-scale pilot purification was conducted using chromatography with cobalt resin, as described in Experimental Procedures. Initially, the elution fraction contained several bands on the BN-PAGE, and only one corresponded to Cho1 (Fig. S3). TEV protease was used to treat the concentrated elution overnight to clear the elution further and cleave the affinity tag. The resulting mixture was again passed over fresh cobalt beads to remove non-specifically bound protein and cleaved tags. Figure 5*A* lane two shows a single 185-kDa protein band on the BN-PAGE after the second affinity chromatography, indicating high purity of the tag-free native Cho1 complex. This complex separated into 30-kDa and 23-kDa bands on the second dimensional SDS-PAGE (Fig. 5*B*), consistent with Figure 3 and the previous findings that *S. cerevisiae* PS synthase separates into 30 kDa and 23 kDa bands on SDS-PAGE (16, 55).

### Enzymology properties and kinetics of *C. albicans* Cho1

**Figure 6:**
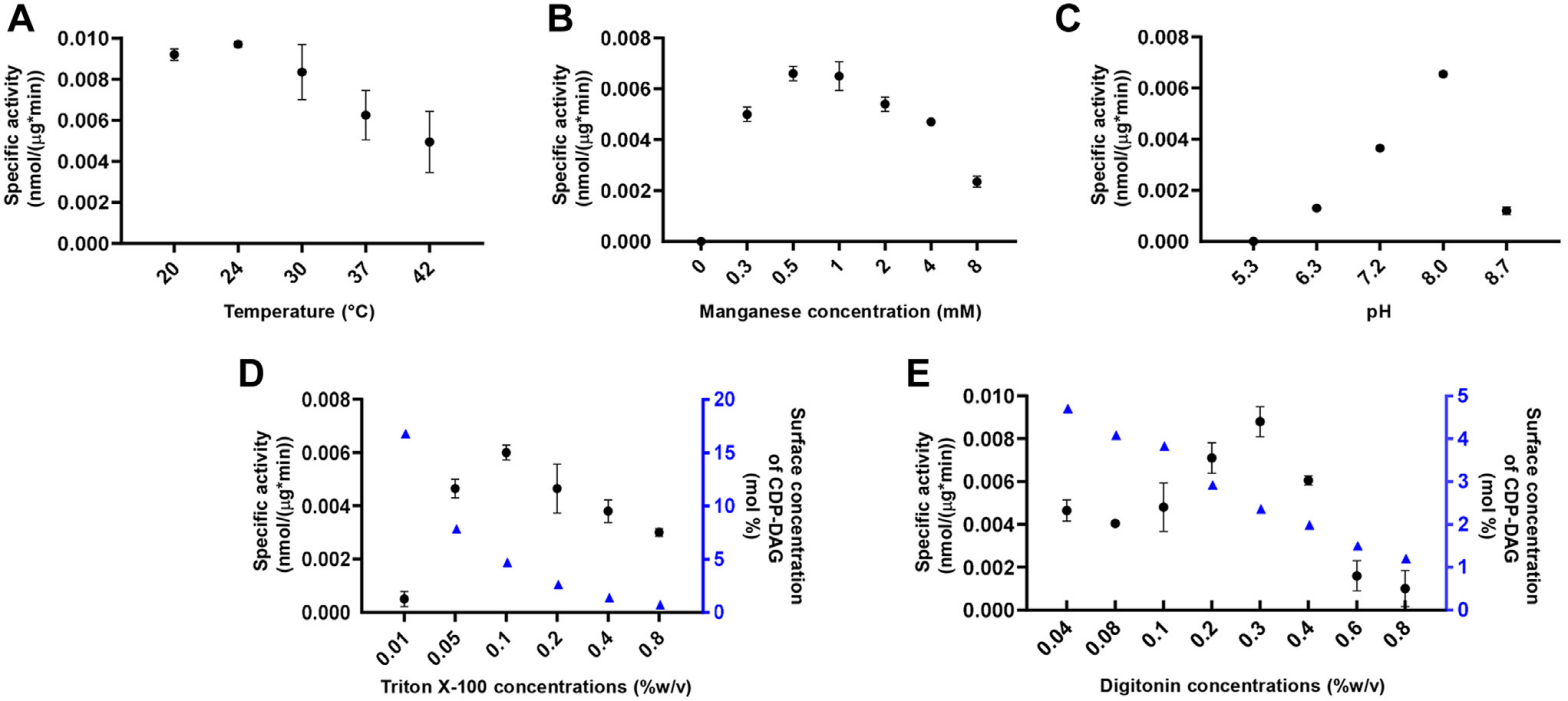
**Effects of temperature, manganese concentration, pH and detergents on purified hexameric Cho1 enzyme activity.** (*A*) temperature, (*B*) manganese concentrations, (*C*) pH, (*D*) Triton X-100 concentrations, and (*E*) digitonin concentrations were varied as indicated, and specific activities (*black filled circle*, nmol/(μg∗min)) of purified Cho1 under each condition were measured as described in the Experimental Procedures. The surface concentrations of CDP-DAG (*blue filled triangle*) in mixed micelles (mol %) were also calculated and shown in (*D*) and (*E*). The specific activities were measured in two biological replicates, and data points are shown as the mean ± S.D. values.

The enzymological properties of the purified Cho1 complex were then tested, and the specific activities were measured in different conditions. The optimal temperature of the Cho1 complex appeared to be 24 °C, although this was not significantly different than activity at 30 °C or 20 °C (Fig. 6*A*). This is surprising since *C. albicans* is a human fungal pathogen and lives in an environment of 37 °C. The optimal manganese cofactor concentration was between 0.5 to 1.0 mM and decreased above 1.0 mM, and the pH optimum is around 8.0 (Fig. 6, *B* and *C*). These enzymological properties of *C. albicans* Cho1 are consistent with those of the *S. cerevisiae* Cho1 homolog previously reported (44). For detergents used in the reaction, Triton X-100 was shown previously to stimulate the activity of *S. cerevisiae* Cho1, and the Triton X-100-CDP-diacylglycerol micelle also serves as a substrate in this reaction (44, 58). *C. albicans* Cho1 activity was tested in different detergent concentrations. Even if the molar concentration of CDP-DAG was unchanged, the addition of detergents to the reaction dilutes the surface concentration of CDP-DAG in a mixed micelle (59), so the mole fraction (mol %) of CDP-DAG was also calculated (Fig. 6, *D* and *E*, blue triangles). By varying the Triton X-100 concentration from 0.01% to 0.8%, we identified the optimal Triton X-100 concentration as 0.1%, and the Cho1 activity decreases at higher concentrations probably due to the drop in CDP-DAG surface concentration (Fig. 6*D*). Besides Triton X-100, digitonin was used as the solubilization detergent, which is also involved in the reaction. Surprisingly, when different concentrations were tested in the reaction, digitonin was found to stimulate the activity of Cho1, even with the decrease in the surface concentrations of CDP-DAG, with the greatest efficiency observed at 0.3% (Fig. 6*E*).

**Figure 7:**
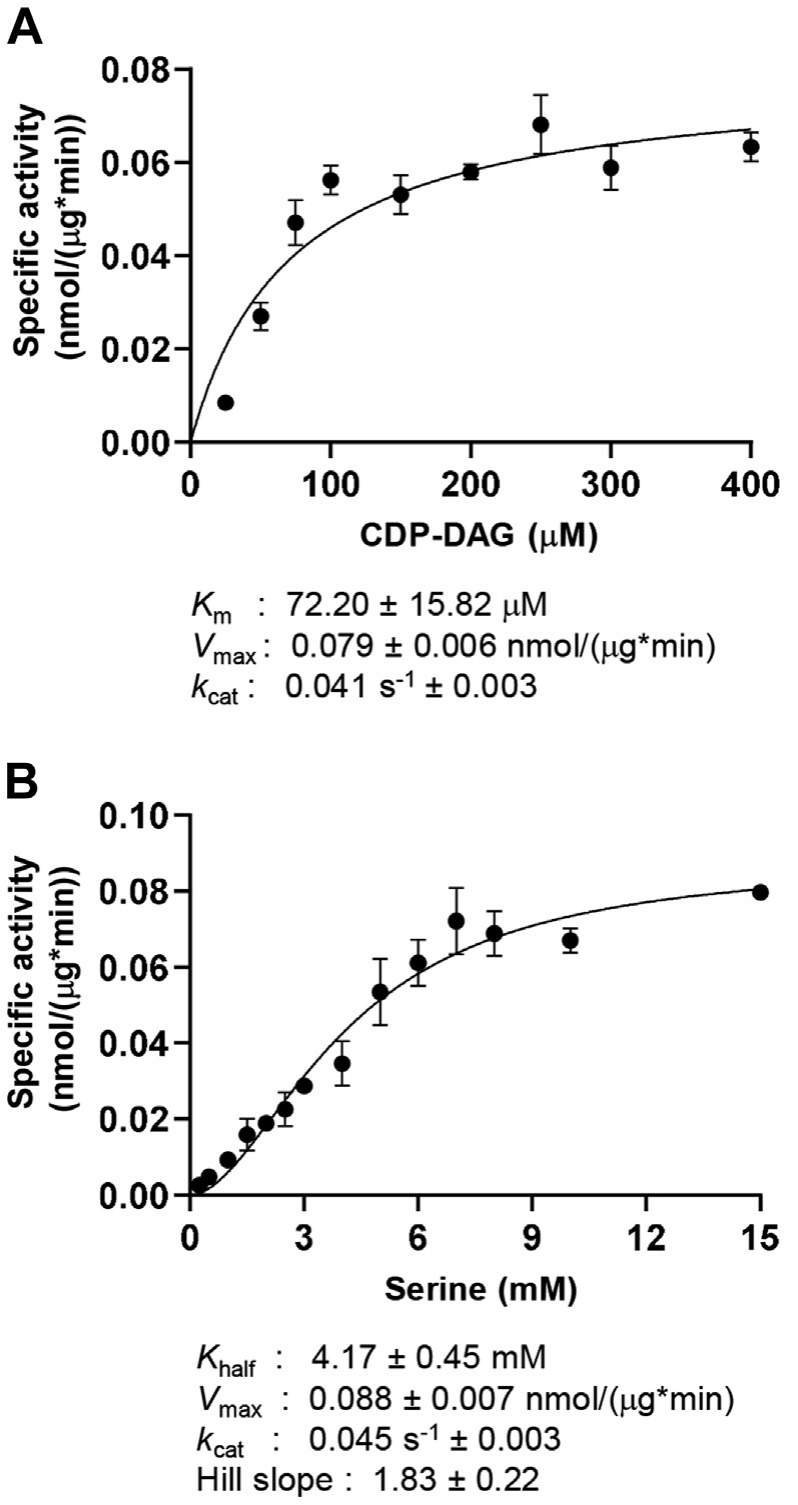
**Kinetic curves suggest a cooperative serine binding in the purified hexameric Cho1.***A*, kinetics curve for CDP-DAG. Serine was kept constant at 19 mM, and the specific activities of purified hexameric Cho1 were plotted against various CDP-DAG concentrations (surface concentration 4.5–5.3 mol %). *B*, kinetics curve for serine. CDP-DAG was kept at 300 μM (surface concentration 5 mol %), and the specific activities of purified hexameric Cho1 were plotted against various serine concentrations. The specific activities were measured in three biological replicates, and data points are shown as the mean ± S.D. values.

In addition, the kinetics of the purified Cho1 complex were measured. For determining the *K*_m_ and *V*_max_ of CDP-DAG, serine was kept constant at 19 mM, and the specific activities of Cho1 were measured at CDP-DAG concentrations of 25, 75, 100, 150, 200, 250, 300, and 400 μM with a surface concentration between 4.5 and 5.3 mol % (Fig. 7*A*). The *K*_m_ for CDP-DAG was estimated at 72.20 ± 15.82 μM, while the *V*_max_ was found to be 0.079 ± 0.006 nmol/(μg∗min). This *K*_m_ is very close to that of the partially purified *S. cerevisiae* PS synthase, which is 60 μM (44). For serine, the CDP-DAG concentration was fixed at 300 μM, and the serine concentration was varied between 0.25 and 15 mM, and the specific activities were measured and plotted against the serine concentrations. This kinetic curve fits better into a sigmoidal curve, indicating some cooperativity in serine binding (Fig. 7*B*). The Hill slope for cooperativity was estimated to be 1.83 ± 0.22 and the *V*_max_ to be 0.088 ± 0.007 nmol/(μg∗min). The *K*_half_, which represents the substrate concentration when the enzyme reaches half of the *V*_max_ in the allosteric sigmoidal substrate-velocity curve, was estimated to be 4.17 ± 0.45 mM. In both cases, the *k*_cat_ is estimated between 0.041 and 0.045 s^−1^.

### *C. albicans* Cho1 is very likely to be a trimer of dimers

To date, all of the experimentally reported structures of membrane-bound CDP-alcohol utilizing phospholipid synthases have been dimers (22, 23, 24, 25, 26, 27). In contrast, data from the BN-PAGE, as well as other MW estimation techniques (Fig. 3), suggest that Cho1 is found in a larger complex. When further examined by SDS-PAGE, high-MW Cho1 contains only bands corresponding to Cho1 (Figs. 3, *A*–*C* and 5), so this complex represents a higher oligomeric state of Cho1, and the molecular weight suggests a hexamer (185 kDa as a complex and 30 kDa as a monomer). However, the organization of this hexamer was unclear.

**Figure 8:**
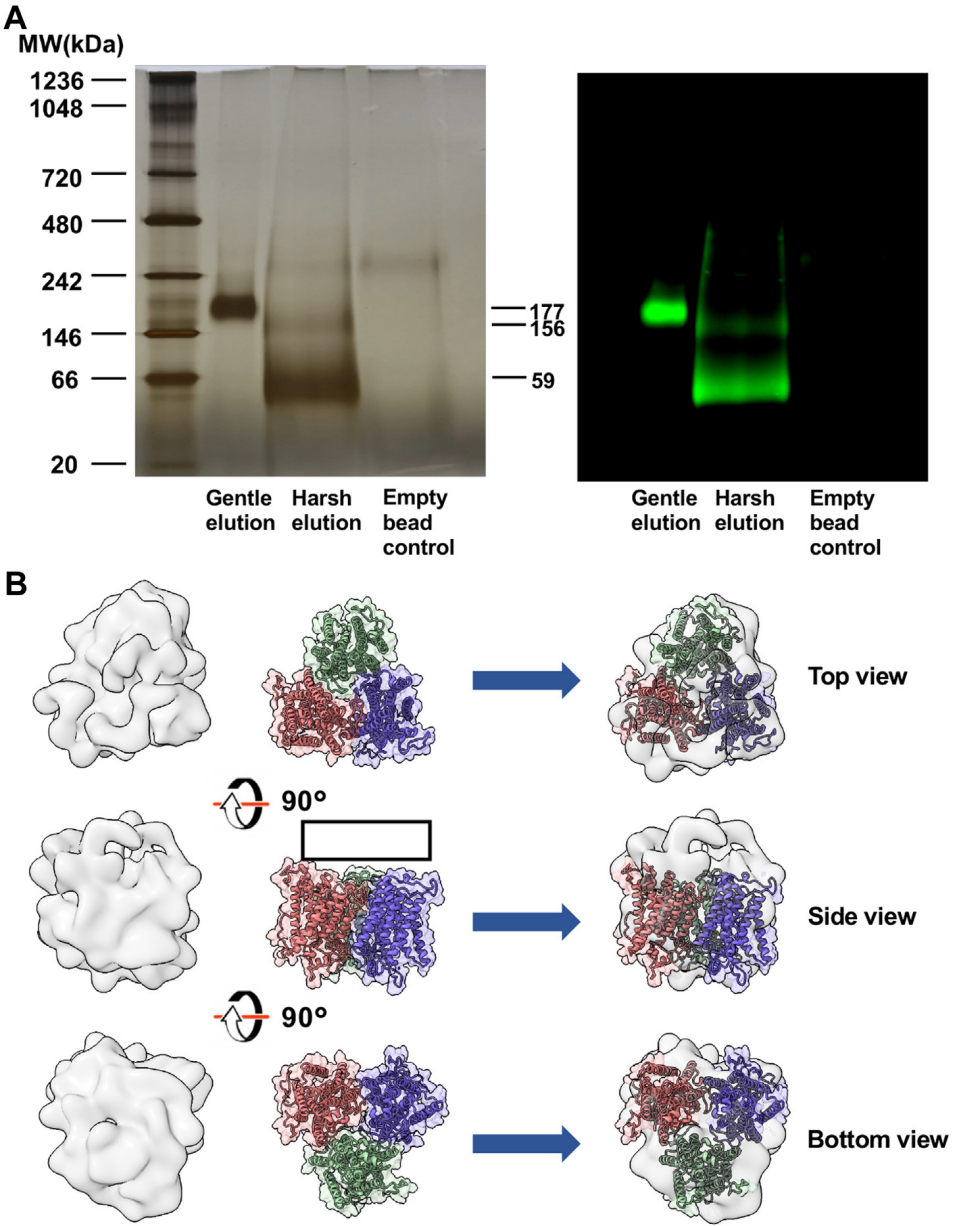
**Hexameric Cho1 is likely made of a trimer of dimers.***A*, BN-PAGE gel (*left*) and a subsequent Western blot of the same gel (*right*) are shown for Cho1 that was eluted from HA protein purification resin with either synthetic HA peptide (gentle elution) or NaOH followed by 1 M TrisHCl pH = 8.5 (harsh elution). A protein-free resin control was eluted using harsh elution. MW of different bands (kDa) were estimated from the protein ladder and indicated. *B*, the *ab-initio* 3D density map of pulled-down Cho1 built from negative staining electron microscopy (*left lane*) fits well with the predicted trimer of Cho1 dimers (*middle lane*) in three different views. A *black box* indicates the region of the deleted 50 amino acid residues from the N-terminus of Cho1.

To test whether the higher oligomer of Cho1 can be broken into smaller oligomers, anti-HA beads were used to pull down the Cho1 complex. This was eluted by either using excess HA peptide or 50 mM NaOH followed by 1 M Tris-HCl (pH = 8.5). HA peptide exchange is a gentle way to elute proteins from anti-HA beads. In contrast, NaOH plus Tris-HCl is a harsh way that also disrupts loose protein associations. The pull-down products from two elution methods and a control group in which the anti-HA beads have no bound proteins and are eluted with NaOH were assessed by BN-PAGE and a subsequent immunoblot. The gentle elution results in a higher protein oligomer (Fig. 8*A*), but the harsh elution leads to two bands on the BN-PAGE, one at 59 kDa and the other at 156 kDa. These bands both correspond with Cho1 protein, as evidenced by immunoblotting (Fig. 8*A*). Since the MW of monomeric HAx3-Cho1 is 36 kDa, the 59 kDa band is likely a dimeric form of Cho1, while the 156 kDa bands represent a trimer of dimers. These results strongly support the native oligomer as a hexamer composed of a trimer of more stable dimers.

To gain insights into the shape of the hexameric Cho1 complex, negative staining electron microscopy and single-particle image analysis were performed on the Cho1 complex following the pull-down. It is well known that detergent micelles present in the sample can produce background noise in the negative staining micrographs and significantly distort the interpretation of small membrane proteins (60). However, the digitonin micelle with an apparent mass of 124 kDa (57) is smaller than the Cho1-digitonin micelle complex (∼300 kDa, Fig. 3), so the larger particles were intentionally collected for analysis. A total of 564,320 particles were picked and 2D classified, resulting in a 2D class average (Fig. S4, *A* and *B*). The best particles, chosen by size and signal, led to five 3D density maps built as described in the Experimental Procedures (Table S1 and Fig. S4*C*). Among the five, density map iv has evident contours and reveals discrete features and thus was selected for analysis (Fig. 8*B*, left lane). In the meantime, a dimeric form of the Cho1 structure was predicted using AlphaFold2 software (Fig. S5*A*). The predicted structure's N-terminal 50 amino acid residues were deleted due to their intrinsically disordered nature and low prediction confidence score (Fig. S5*B*).

A predicted trimer of the dimeric Cho1 complexes was simulated by the HSYMDOCK docking web server (Fig. 8*B*, middle lane) (61). It was then fitted with the 3D density map generated from the negative staining electron microscopy (Fig. 8*B*, right lane). As shown, the 3D density map fits well with the predicted trimer of Cho1 dimers in the top and side views, given that the N-terminal region of Cho1 was omitted in the predicted trimer-of-dimers structure (Fig. 8*B*, right lane). The bottom perspective shows additional density in the 3D map absent in the trimer-of-dimers structure, which can be explained by the bound digitonin micelle and residual bound phospholipids. In sum, the 3D density map generated here is believed to represent the overall shape of a trimer of Cho1 dimers in a complex.

## Discussion

Here, we have developed and optimized solubilization and purification methods for the membrane-bound Cho1 protein from *C. albicans*. Several detergents were used to solubilize Cho1, and digitonin stands out among them, as the Cho1 protein was purified in a single conformation after being solubilized by digitonin (Figs. 3*A* and 5A). However, it is noticeable that the digitonin-solubilized Cho1 complex has a higher MW than Cho1 solubilized by other detergents (Figs. 1*A* and 2, *A* and *C*). It is possible that this difference is due to Cho1 being solubilized in a different conformation or being found within a different lipid-detergent micelle. However, another possibility is that digitonin might conserve the interaction between Cho1 and an unknown protein(s) in the crude solubilization fraction due to the very mild solubilization property of digitonin (49, 62). This is supported by the observation that in digitonin, the solubilized Cho1 complex from the crude fraction has a higher MW than Cho1 from pull-down reactions (216 kDa *versus* 172 kDa, Figs. 1*A* and 3A). The decrease in MW (∼44 kDa) during the pull-down process could represent a loss of the unknown protein(s). The interaction(s) between Cho1 and unknown proteins in the crude fraction may not be preserved by other detergents and may be lost even for digitonin during the purification process.

Another advantage of digitonin is that it not only retains but also stimulates the enzymatic activity of Cho1 (Figs. 2*B* and 6E). This is interesting because, unlike Triton X-100, which was used to dissolve the substrate CDP-DAG and was previously shown to provide a surface for catalysis (58), digitonin served to solubilize Cho1 and keep Cho1 in the micelle. In contrast, the synthetic substitute of digitonin, GDN, despite having a similar steroid-based structure as digitonin (63, 64, 65), failed to maintain PS synthase activity after solubilization (Fig. 1, *B* and *C*). Currently, we do not have an explanation for the stimulant effect of digitonin, and further experiments are required to explain this stimulant effect.

Since *C. albicans* Cho1 uses CDP-DAG as one of the substrates and has the CAPT motif, it belongs to the CDP-AP family (18). So far, six protein members within this family have solved crystal structures, all of which are dimers (22, 23, 24, 25, 26, 27). Here, we show for the first time that *C. albicans* Cho1 forms hexamers based on MW from BN-PAGE (Figs. 3 and 5). It has previously been shown that the migration of solubilized proteins on BN-PAGE can be affected by the choice of detergent, detergent concentration, and micelle lipid composition, thus skewing the MW estimation (49). To account for this, six different non-ionic detergents, both mild and harsh, were used to solubilize Cho1, which led to protein bands of a similar high MW for Cho1 and suggested hexamers in each case (Fig. 1*A*). Also, the concentration of digitonin and DDM varied from 0.5% to 1.5% and 0.5% to 2.1%, respectively, which will change the micelle lipid compositions, but only single bands corresponding with hexamers appeared in the BN-PAGE (Fig. 2, *A* and *C*). In addition, size-exclusion chromatography, mass photometry, and analytical ultracentrifugation have also indicated the MW of native Cho1 protein alone is 180 kDa (Fig. 3, *D*–*F*), once the micelle MW is subtracted. These results taken altogether suggest that Cho1 forms hexamers.

Furthermore, a harsh elution of Cho1 from the anti-HA beads with NaOH and 1 M Tris-HCl indicated that the hexamer species is made of Cho1 dimers (Fig. 8*A*). Thus, we have concluded that Cho1 forms a trimer-of-dimers in the native environment (Fig. 8). The SMALP 2000 solubilization fraction further supports this, producing both a hexamer and a dimer based on the MW (Fig. 2*E*). Since SMALP 2000 solubilizes proteins in the native environment, this points to the existence of both Cho1 hexamers and dimers in the native membrane, with the hexamer being more abundant. We have further shown the likely presence of a simulated trimer of Cho1 dimers in the sample *via* negative staining electron microscopy (Figs. 8*B* and S4). Together, those results suggest that *C. albicans* Cho1 forms hexamers and is likely a trimer-of-dimers. The explanations for the oligomer state discrepancy between *C. albicans* Cho1 and structures of other CDP-APs include: (i) the loose association between three Cho1 dimers may be broken during the crystallization process, or (ii) the eukaryotic *C. albicans* Cho1 has evolved a higher oligomer state compared to the six solved prokaryotic counterparts.

Finally, a surprising finding is the sigmoidal velocity-substrate concentration curve for serine, indicating that Cho1 cooperatively binds serine. Both high and low *K*_m_ values have been reported previously for both crude and purified *S. cerevisiae* PS synthase (42, 44, 66, 67), and Kiyono *et al.* (17) observed a serine *K*_m_ of 0.14 mM at low L-serine concentrations and 9.5 mM at high L-serine concentrations in both microsomal and partially purified *S. cerevisiae* PS synthase. All those results together suggest potential cooperativity in the serine-binding of PS synthase. We do not know whether cooperativity occurs within one protomer or between protomers. Interestingly, two serine-binding pockets were identified in the PS synthase structure of *M. jannaschii* (Fig. S6). Still, the authors later considered one serine-binding pocket (Fig. S6, serine 1) an experimental artifact due to the less optimal position for reaction (23). However, binding the less optimal serine likely facilitates binding the optimal serine for catalysis. Thus, the cooperative serine binding is present within one protomer. However, since *M. jannaschii* PS synthase is the first and only PS synthase with a solved structure, and due to the distant phylogenic relationship between *M. jannaschii* and *C. albicans*, there is limited information available, and a solved *C. albicans* Cho1 structure will give insight into this question. Here, we have developed the solubilization and purification methods for hexameric *C. albicans* Cho1, which can be used for small molecule screening or structure determination.

## Experimental procedures

### Strain construction and media

This study used the HA1 *C. albicans* strain to solubilize Cho1. This strain was previously created and expressed a HAx3-tagged Cho1 protein from a strong constitutive promoter, as described in (18). The yeast strains constructed in this study are also listed in Table 1. Media used in this study include YPD (1% yeast extract, 2% peptone, 2% dextrose) and minimal medium (0.67% yeast nitrogen base, 2% dextrose ± 1 mM ethanolamine).

**Table 1:** Strains used and produced in this study

Organism	Strain	Plasmid	Genotype
*Candida albicans*	HA1	pCDC4	*cho1ΔΔ*_*PENO1*_*-CHO1-HAx3*
*Candida albicans*	YZ81	pYZ79	*cho1*ΔΔ*P*_*ENO1*_*-CHO1-ENLYFQG-HAx3-Hisx8*
*Candida albicans*	YZ88	pYZ84	*cho1*ΔΔ*P*_*ENO1*_*-CHO1- ENLYFQG*-*HAx3-GST*
*Candida albicans*	YZ89	pYZ85	*cho1*ΔΔ*P*_*ENO1*_*-CHO1- ENLYFQG-HAx3-MBP*

To create new constructs, site-directed mutagenesis was performed on the plasmid pCDC4 (18), harboring gene *CHO1-HAx3*, to generate a TEV protease recognition site (ENLYFQG) between the *CHO1* and *HAx3* gene using a primer-based method (Table 2, YZO 67&68). The *CHO1-ENLYFQG-HAx3* gene was PCR-amplified with a forward primer (YZO 64) and a reverse primer (YOZ 66) that added a Hisx8 tag at the end of the existing HAx3 tag. The following amplification product was then cloned into the plasmid pBT1, which contains the constitutive *ENO1* promoter (*P*_*ENO1*_) and the *SAT1* marker (68), to create the plasmid pYZ79. *C. albicans* codon-optimized GST and MBP genes purchased from Genscript were fused to the *CHO1-ENLYFQG-HAx3* gene by replacing the existing Hisx8 tag in pYZ79 *via* amplification by PCR with the appropriate primers (Table 2) to create plasmids pYZ84 and pYZ85, respectively. Plasmids pYZ79, pYZ84, and pYZ85 were linearized with *MscI* restriction enzyme (within the *P*_*ENO1*_ sequence) and then were electroporated into the *cho1*ΔΔ *C. albicans* strain (11). The transformants were selected on the YPD plates containing 100 μg/ml nourseothricin. Colony PCR was performed on six candidates for each gene construct to ensure the successful integration under the *P*_*ENO1*_ promotor on the chromosomal DNA, and no spurious mutations occurred during the transformation.

**Table 2:** Primers used in this study

Primer name	Sequence	Function/Mutation
YZO 67	atctcaaaatctttaaaaattcctaaaccagcggccgtgaaaattttattttcaaggttcatacccatacgatgttcctgactat	Adding TEV protease recognition site (ENLYFQG)
YZO 68	ggatcctgcatagtccgggacgtcatagggatagccggcatagtcaggaacatcgtatgggtatgaaccttgaaaatataaattttcaac	Adding TEV protease recognition site (ENLYFQG)
YZO 64	aaaagcggccgcatgtcagactcatcagctaccgggttctccaagcaccaagagtcagcaattgtatcagattcagaaggag	Adding Hisx8 tag to the existing HA tag
YZO 66	aaagagctcctatccaccgtgatggtgatggtgatggtgatgggcggccggagcgtaatctggaacgtcatatggataggatcctgcatagtccgggacg	Adding Hisx8 tag to the existing HA tag
YZO 69	tatccctatgacgtcccggactatgcaggatcctatccatatgacgttccagattacgctccggccgccgttatgtcccctatactaggttattgg	Replacing the existing Hisx8 tag with a GST tag
YZO 70	ttttgagctcctatccaccttttggaggatggtcgccaccacca	Replacing the existing Hisx8 tag with a GST tag
YZO 71	tatccctatgacgtcccggactatgcaggatcctatccatatg	Replacing the existing Hisx8 tag with an MBP tag
YZO 72	ttttgagctcctatccacccgtttctcgttcagcttttttgta	Replacing the existing Hisx8 tag with an MBP tag

### Spot dilution assay and growth curves

Spot dilution assays and growth curves were conducted as described in (18).

### PS synthase assay

The PS synthase assay was used to measure the enzymatic activity of the Cho1 protein. Cells were lysed as previously described to isolate membranes containing PS synthase using a French press (45). Then the crude membranes were collected by centrifugation at 3,000*g* to remove intact cells. The remaining supernatant was centrifuged at 27,000*g* for 30 min to pellet the cellular membranes. The reaction was then set up as described in (18), with the adjustment of 30 °C as the reaction temperature. Briefly, PS synthase activity was measured by counting the total radioactivity of L-[^3^H]-serine in the chloroform phase (phosphatidylserine), normalized to the protein amount (0.5 mg crude membrane protein) as measured by the Bio-Rad protein assay and dividing it by the reaction time (30 min). The reagents were added to final concentrations of 50 mM Tris-HCl (pH = 8.0), 1 mM MnCl_2_, 0.1% Triton X-100, 0.1 mM CDP-DAG, 0.5 mM L-serine (spiked with 5% (by volume) L-[^3^H]-serine (30 Ci/mmol)), 5% glycerol and 1 mM BME, at a total volume of 100 μl. For the solubilized fractions, 50 μl volume was used in each reaction, and the protein concentration of each solubilized fraction was measured using the Pierce detergent compatible Bradford assay kit. To calculate the adjusted PS synthase activity, PS synthase activity was normalized to the relative densitometry values of the Cho1 bands from each sample run on a Western blot as described in (18) and expressed as nmol/(μg protein∗min). The specific activity was measured using the same conditions (unless otherwise indicated) with 1 to 2 μg purified Cho1 protein. Specific activity was calculated based on the slope of linear PS production, from at least three time points, within 30 min, representing the initial velocity.

### Western blot

Western blots of the Cho1 protein fractionated on SDS-PAGE gels were conducted as described in (18). PVDF membranes were used for BN-PAGE gels, and the wet transfer was done in 1X NuPAGE Transfer buffer at 25 V and 4 °C for 1 h. The subsequent membrane was de-stained in 100% methanol before blotting. The blotting procedure is the same as the SDS-PAGE membrane described in (18).

### Cell lysis, solubilization, and purification

*C. albicans* strains were grown in YPD to OD_600_ between 6.5 to 8.0 for solubilization and purification. The cells were centrifuged and lysed, as previously described (45). Cell lysates used for large-scale purification were supplemented with 10 μg/ml leupeptin, pepstatin A, and 10 μM PMSF. The crude membranes were clarified by centrifugation at 3000*g* for 10 min to remove unbroken cells. Then the supernatant was recentrifuged at 100,000*g* for 60 min to pellet the cellular membranes. The crude membranes were resuspended in 50 mM Tris-HCl (pH = 8.0), 2 mM MnCl_2_, 30 mM MgCl_2_, 10 mM KCl, 10% glycerol, and 2 mM BME to a final protein concentration of 10 mg/ml as measured by Bio-Rad, protein assay dye before solubilization by detergents. For SMA solubilization, the crude membrane was resuspended in a modified buffer: 50 mM Tris-HCl (pH = 8.8), 125 mM KCl, 10% glycerol, and 10 mM BME. Detergents or SMAs (Cray Valley) were dissolved in H_2_O and added to the crude membrane prepared to the final concentration indicated. The solubilization was carried out at 4 °C (for detergents) or 37 °C (for SMAs) for 60 min or the time otherwise indicated, which was then cleared through centrifugation at 100,000*g* for at least 30 min. The subsequent supernatant was considered the solubilized fraction.

Protein pull-downs were performed with Pierce anti-HA magnetic beads. Following solubilization, 50 μl of anti-HA magnetic beads were applied to the solubilization fraction and gently mixed for 30 min at room temperature. The beads were washed twice with 500 μl TBS + 0.1% digitonin, followed by a final wash of H_2_O + 0.05% digitonin. The bound protein was eluted using a solution of 2 mg/ml HA synthetic peptide (Thermo Scientific) + 0.05% digitonin for gentle elution. For harsh elution, 100 μl of 50 mM NaOH + 0.05% digitonin buffer was used, followed by adding 50 μl 1 M Tris, pH 8.5 + 0.05% digitonin.

Protein column purification was performed using HisPur cobalt resin (Thermo Scientific). Briefly, HisPur Cobalt Resin was prepacked on the chromatography column and washed with 10 bead-bed volumes of washing solution (50 mM Tris-HCl (pH = 7.0), 15 mM imidazole, and 0.1% digitonin). Solubilized fractions, from 1% digitonin or other indicated conditions, were gently loaded and run through the pre-washed beads *via* gravity. The flow-through fraction was again loaded on the column twice for maximum binding. Then at least six column volumes of washing solution were used to wash out non-specifically bound proteins. Elution was performed using 6× column volumes of the elution buffer (50 mM Tris-HCl (pH = 8.0), 150 mM imidazole, and 0.05% digitonin or other detergents). The elution fraction was concentrated to 500 μl and exchanged with 4 ml dilution buffer (50 mM Tris-HCl (pH = 8.0), 0.1% digitonin) in the Pierce Protein Concentrator PES (10K MWCO, 5–20 ml) twice, and again concentrated to ∼500 μl final volume. The protein concentration of the final buffer-exchanged sample was measured using the Pierce detergent-compatible Bradford assay kit. To cleave the His-tag, AcTEV protease (Invitrogen) was applied at the ratio of 1 μl: 30 μg protein of the buffer-exchanged sample in the presence of 2 mM DTT. The cleavage reaction was carried out overnight at 4 °C. Following cleavage, the sample was applied to fresh pre-washed HisPur cobalt resin to remove any non-specifically bound protein, free His-tags, and the uncleaved Cho1 protein. The final protein concentration was measured using the Pierce detergent compatible Bradford assay kit, and the cleaved protein was used for specific activity determination.

### BN-PAGE and second-dimensional SDS-PAGE

The NativePAGE system was used for the BN-PAGE. 0.5% to 1% NativePAGE G-250 sample additive, 1× NativePAGE Sample Buffer, and 2 μg solubilized protein (2 μl for SMA-solubilized fractions and 14 μl for purified protein) were mixed and loaded on the NativePAGE 4 to 16%, Bis-Tris gel. NativeMark unstained protein standard (Invitrogen) was used as the protein ladder for MW estimation. The gel was run at 4 °C with pre-chilled buffers, with NativePAGE dark blue cathode buffer at 70 V for 50 min and NativePAGE light blue cathode buffer at 150 V till the dye reached the gel front. The gels were then analyzed by Western blot (as mentioned above), Coomassie Blue R250 staining, or silver staining.

The second dimensional SDS-PAGE was performed using the Novex system. Briefly, the gel strip was cut from the first dimension NativePAGE gel and equilibrated in the 1× NuPAGE LDS sample buffer with 50 mM DTT for at least 30 min. The gel strip was then transferred to an alkylating solution containing 1X NuPAGE LDS Sample Buffer with 50 mM N, N-Dimethylacrylamide, rocking for 30 min. The gel strip was then transferred to the quenching solution with 1× NuPAGE LDS Sample Buffer with 5 mM DTT and 20% ethanol. Then, the prepared gel strip was mounted to the 2D well of Tris-Glycine Novex 4 to 20% mini protein gel and run at 110 V till the dye reached the gel front. For analysis, the resulting 2D gel was subjected to Coomassie Blue R-250 or silver staining.

### Size-exclusion chromatography

Pull-downed Cho1 was subjected to size-exclusion chromatography. All buffers and samples were filter through 0.22 μM filters. A Superdex 200 10/300 Gl column (Cytiva) was attached to an NGC chromatography system Quest 10 plus (Bio-Rad) for the analysis. To equilibrate the column, 1.5 column volumes of water followed by 1.5 column volumes of elution buffer (50 mM Tris-HCl (pH = 8.0) + 0.04% digitonin) were run through the column at a flow rate of 0.5 ml/min. The pull-downed Cho1 sample or gel filtration standards (Bio-Rad, #1511901) were then manually injected in the sample loop. The elution buffer was used to elute the injected sample at a flow rate of 0.4 ml/min. Fraction collection was enable from 0.16 to 1 column volumes and each fraction had 0.3 ml. Fractions with peaks revealed by the A280 detection were analyzed by SDS-PAGE and immunoblotting.

### Sedimentation velocity analytical ultracentrifugation

Sedimentation velocity experiments were carried out in a Beckman XL-I analytical ultracentrifuge using an An-60Ti rotor. Sample volumes of 400 μl were loaded into charcoal-filled Epon double-sector cells with a 12 mm path length. Samples were equilibrated for 1 h at 20 °C prior to the run. Other experimental conditions included a rotor speed of 50,000 rpm and a temperature of 20 °C. Sedimentation was monitored using the interference optical system scanning every 2 min. Data were fit to a continuous [c(s)] distribution model using SEDFIT (version 16.1c) (69). The protein partial specific volume, buffer density, and buffer viscosity were calculated using SEDNTERP (version 3.0.4) (70).

### Mass photometer

Mass photometry experiments were performed on a Refeyn One^MP^ using the pull-downed Cho1 in the 50 mM Tris-HCl (pH = 8.0) + 0.05% digitonin buffer. Each experiment consisted of 6,000 frames collected over 60 s. Data analysis was carried out using DiscoverMP (version 2.5.0, Refeyn). Mass photometry contrast was converted to mass values using a standard curve generated using BSA (monomer), BSA (dimer) and thyroglobulin (dimer).

### Electron microscopy

First, 5 μl of the pulled-down sample with a total protein concentration of 0.03 mg/ml was applied to the glow-discharged (PELCO easyGlow, 15 mA, 25 s) Quantifoil 100 Carbon Support Films grid: Cu 300 and left on the grid for 45s (Table S1). The excess sample was removed by blotting. Then, 5 μl of 2% uranyl acetate was applied to the grid and was rapidly removed by blotting after 30 s. Next, the washing step included the application of 5 μl 2% uranyl acetate on the grid and removed immediately. After drying out, the grids were loaded in the 200 kV Thermo Scientific Glacios using an autoloader under cryo-EM conditions.

### Electron microscopy data acquisition

Image acquisition was performed on the 200 kV Thermo Scientific Glacios in bright field imaging mode. Movies were recorded using a Thermo Scientific Falcon 3EC Direct electron Detector in linear mode at a nominal magnification of 92,000, corresponding to a pixel size of 1.567 Å/pix with eight frames at a dose of 30 e−/Å^2^ per frame and an exposure time of 30 s per movie (Table S1).

### Image processing

The dataset of Cho1 protein images was derived from 4,946 movies. The raw movie files were imported into SCIPION 3.0 (71) for the following processing steps. The movies were motion corrected using the MotionCor2-1.4.7 protocol (72), and the CTF estimation was done by the Gctf-1.06 protocol (73). A total of 574,190 particles were extracted after manual/auto Xmipp particle picking. After multiple rounds of 2D classification using the cryoSPARC v.3.2 protocol (74), the main protein complex with apparent features was sorted. The particles for the defined 2D classes were subjected to the cryoSPARC v.3.2 *ab initio* protocol with five categories. Then, the 3D Heterogeneous refinement using five *ab initio* 3D maps obtained at the previous step was followed using cryoSPARC v.3.2. The best-resulting 3D class containing 54,899 particles was subjected to a 3D non-uniform refinement (74). As a result, a final 3D map was obtained. The resulting 3D map underwent rigid fitting using a structure from AlphaFold2 prediction software (75) in ChimeraX (76).

### Statistical analysis and MW estimation on the gels

All the statistical analyses were performed with GraphPad Prism 9.1 software. The PS synthase activities were compared using Brown-Forsythe and Welch ANOVA tests (unequal SDs). All MW estimates were conducted in the band analysis tool of the Quantity One software (Bio-Rad).

## Data availability

The original contributions presented in the study are included in the article/Supplementary Material. Further inquiries can be directed to the corresponding author.

## Supporting information

This article contains supporting information.

## Conflict of interest

The authors declare that they have no known competing financial interests or personal relationships that could have appeared to influence the work reported in this paper.
