# Supplementary material for: Solubilization, purification, and characterization of the hexameric form of phosphatidylserine synthase from Candida albicans
Source: J Biol Chem. 2023 Apr 26;299(6):104756. doi: 10.1016/j.jbc.2023.104756 (PMC10248529; doi:10.1016/j.jbc.2023.104756)

Figure S1. HAx3-tagged Cho1 protein was solubilized in SMA1000, 2000 and 3000 at 2%, 3% and 4% for 2 hours, and the resulting solubilized fractions were detected by Western blotting following BN-PAGE.

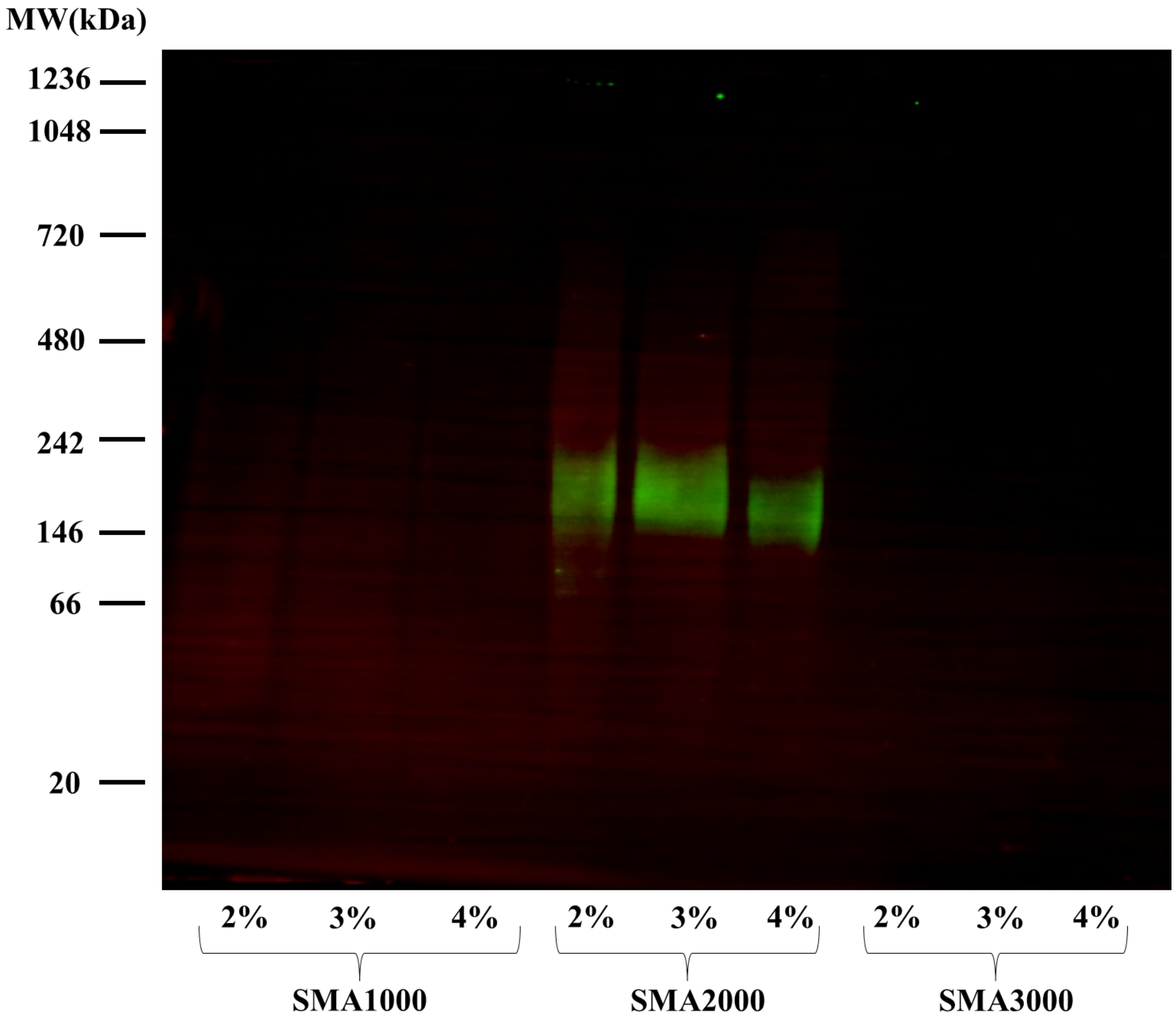

Figure S2. (A) BN-PAGE of the pulled-down HAx3-tagged Cho1 protein from the solubilized fractions of 0.9%, 1.1%, 1.3%, 1.5% DDM. **(B)** The 2nd dimensional SDS-PAGE of the BN-PAGE gel strip from 1.1% DDM. All gels were stained with Pierce™ silver stain kit, and MW of different bands (kDa) were estimated from protein ladders and indicated.

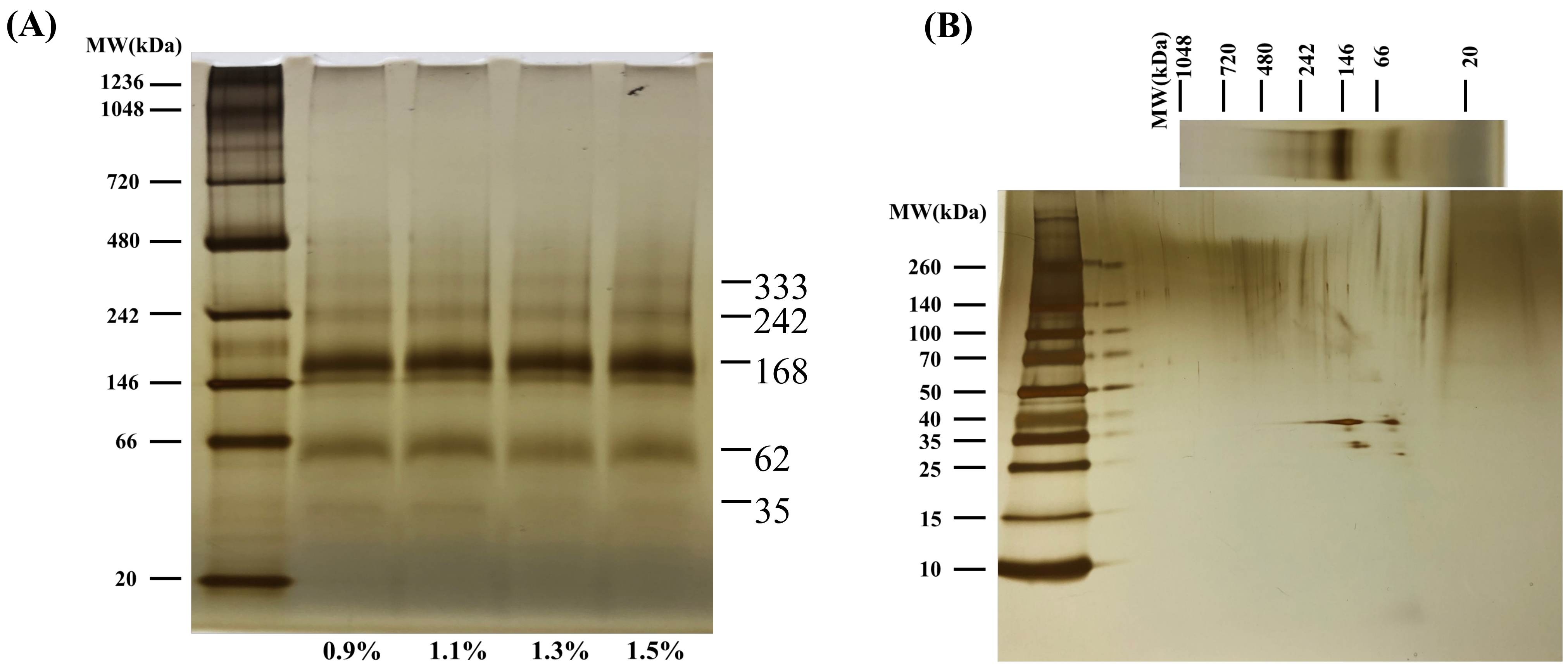

Figure S3. (A) BN-PAGE gel of cobalt resin flowthrough (FT), washes 1, 2, & 3, and elutions 1, 2, & 3 and the concentrated combined elution fractions (Conc. Elution), all stained with Coomassie blue R250 dye. **(B)** Western blotting of the concentrated elution on the BN-PAGE using anti-HA antibody.

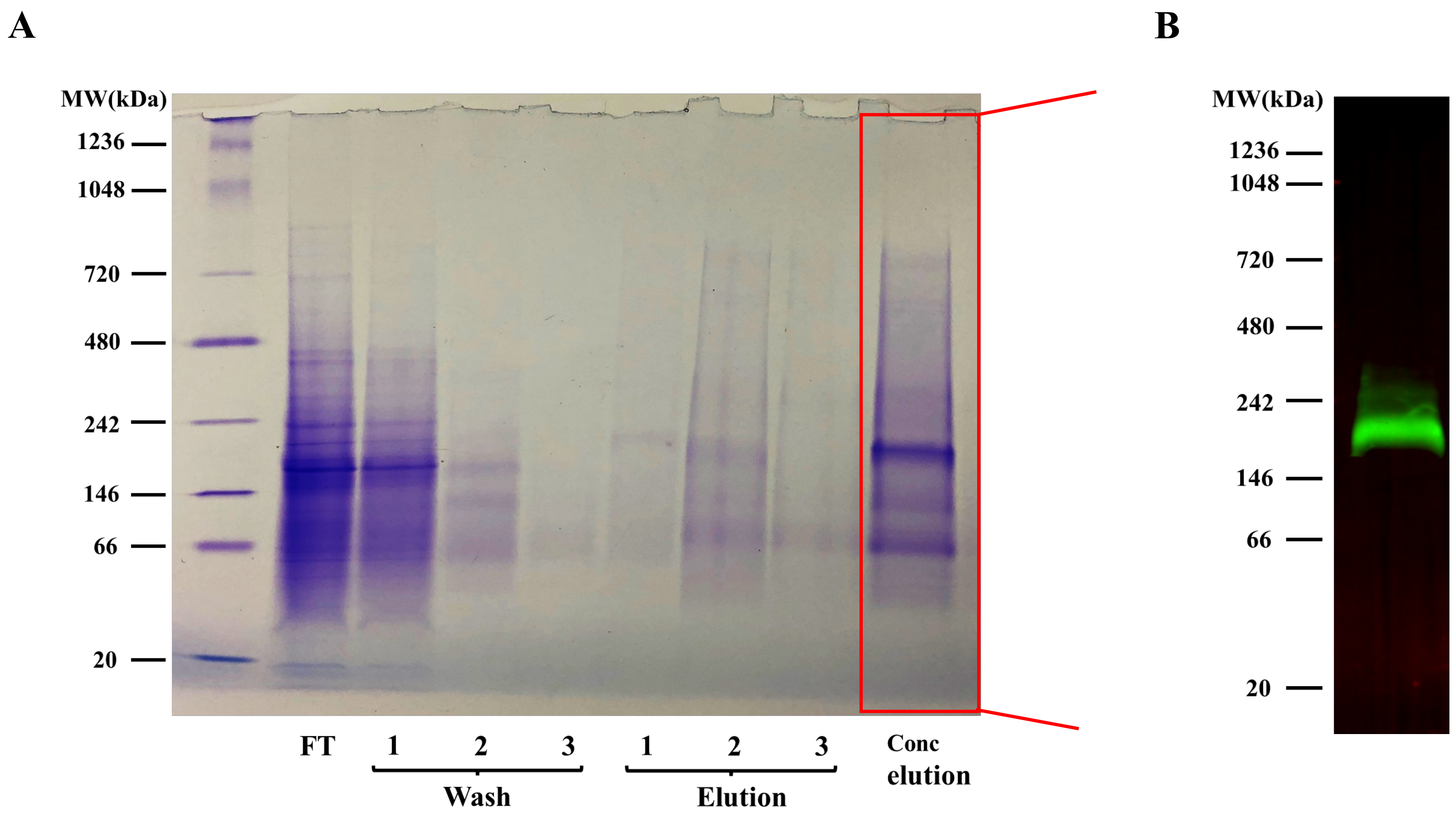

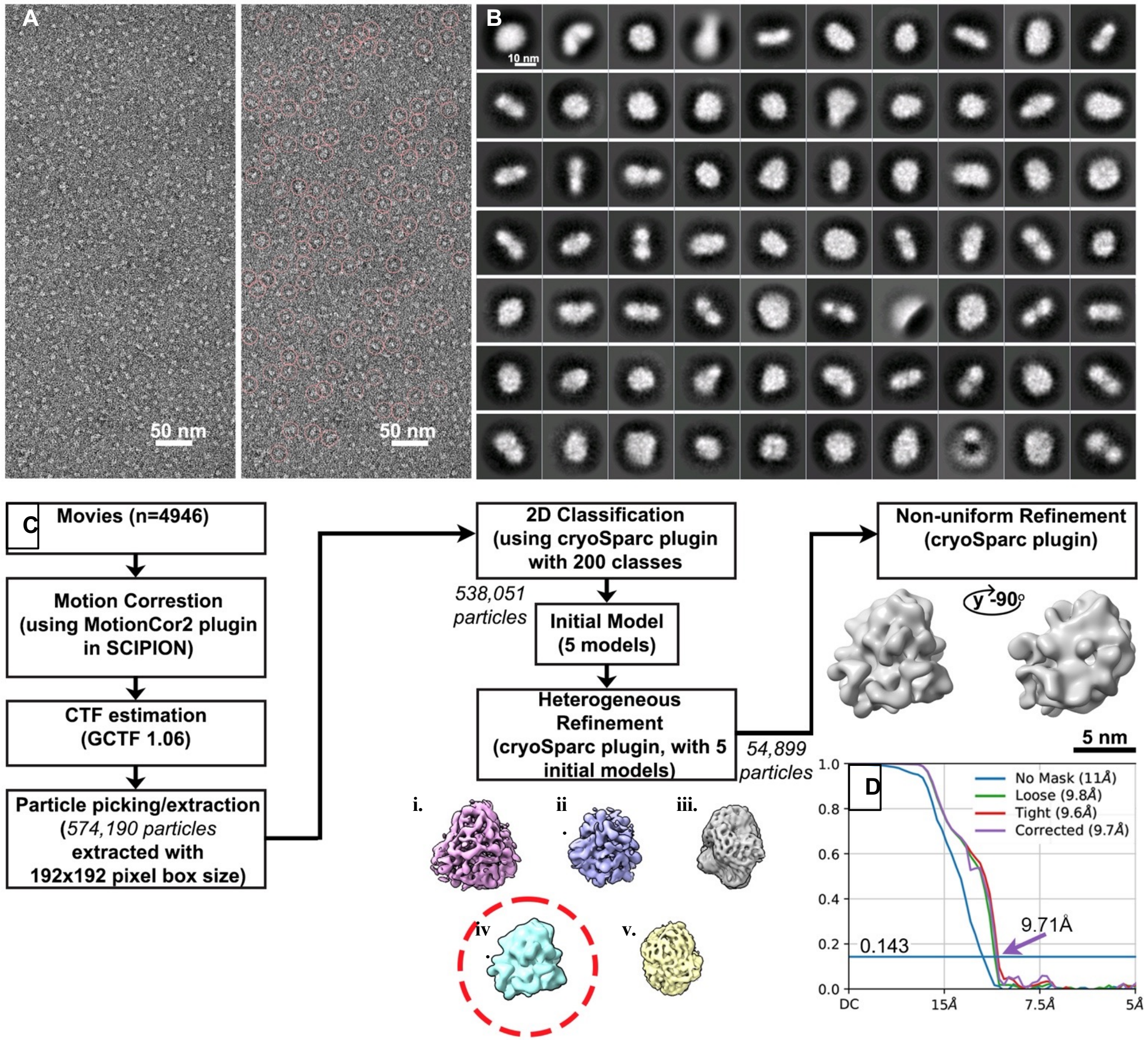

Figure S4. Data processing pathway for the negative staining TEM of the Cho1 micelles
(A) Representative negatively stained TEM micrographs (4,946 in total) of the Cho1 complex (left). All particles picked from that micrograph are marked with pink circles (right). (**B**) Representative reference-free 2D class averages. **(C)** Overview of the negative staining TEM workflow for the Cho1 complex dataset. Red circle shows the 3D model that was chosen for further analysis. **(D)** The gold-standard Fourier Shell Correlation (FSC) curve. Based on the FSC=0.143 criterion, the resolution of the full map is 9.7 Å.

Figure S5. The structure prediction model of the Cho1 dimer using AlphaFold2 **(A)**, and the refined Cho1 dimer with the deletion of N-terminal 50 amino acids is shown in **(B)**.

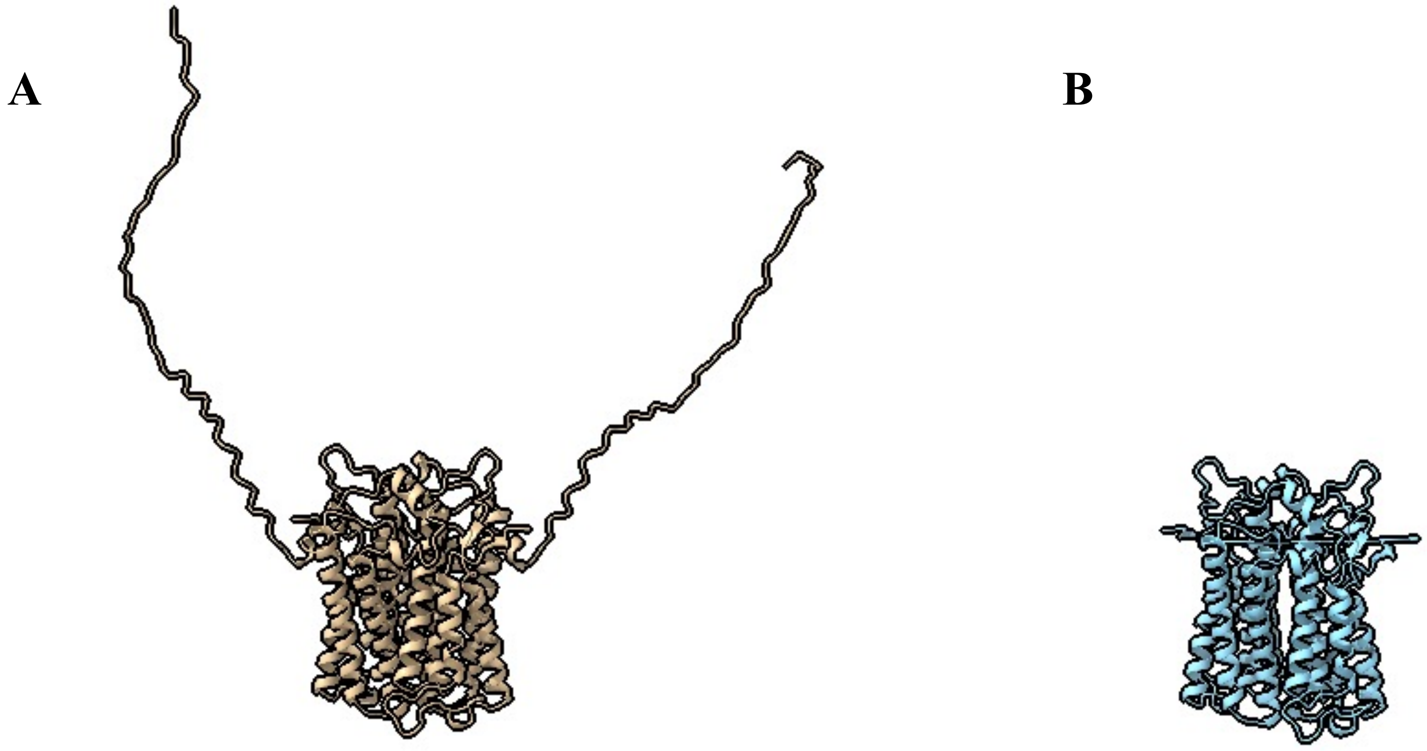

Figure S6. Superposition of AlphaFold2-predicted Cho1 structure (red) with *Methanocaldococcus jannaschii* PS synthase structure (blue, PDB: 7B1L) at two different views. The conserved CAPT motif was shown as green in the predicted Cho1 structure and yellow in *Methanocaldococcus jannaschii* PS synthase structure. The two bound serines are from *Methanocaldococcus jannaschii* PS synthase structure. Serine 1 was claimed to be an experimental artifact.

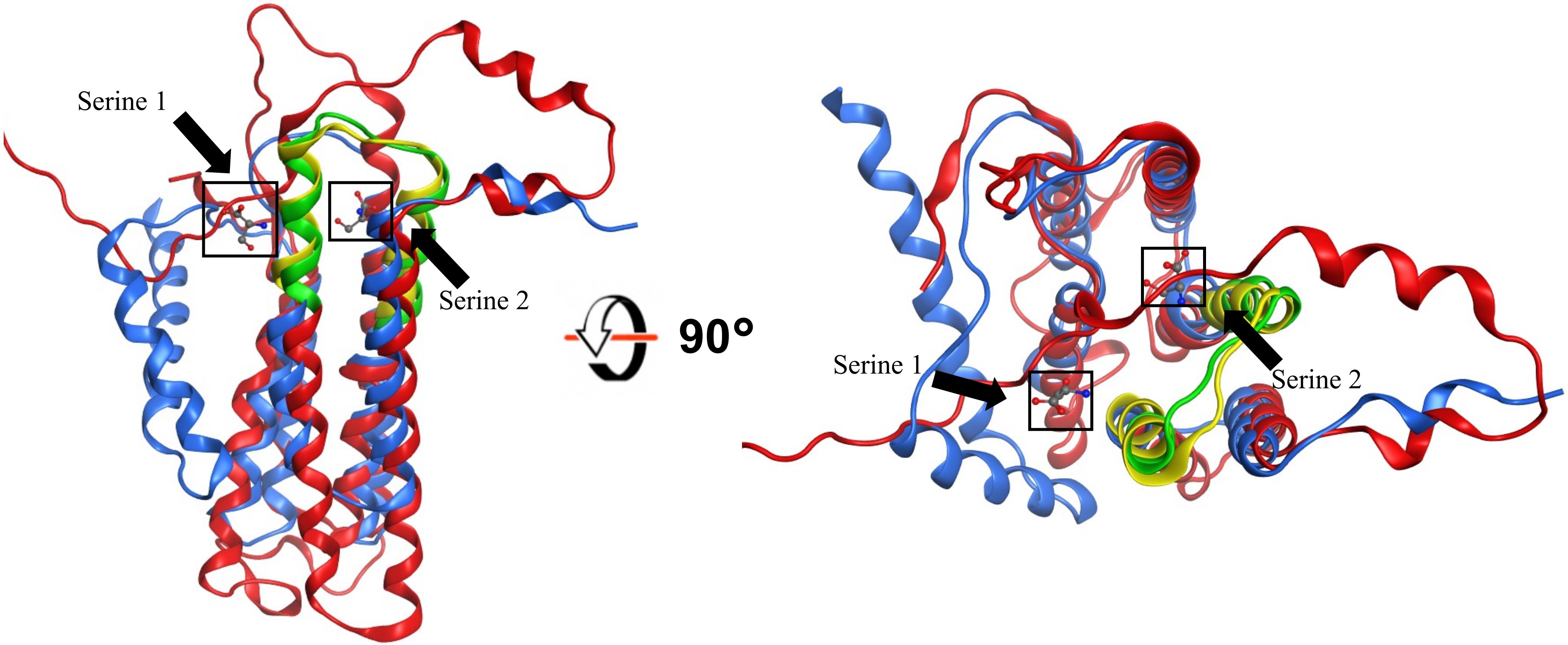

Supplement: Supporting Figures S1–S6 [file mmc1.pdf]
