# Supplementary material for: Solubilization, purification, and characterization of the hexameric form of phosphatidylserine synthase from Candida albicans
Source: J Biol Chem. 2023 Apr 26;299(6):104756. doi: 10.1016/j.jbc.2023.104756 (PMC10248529; doi:10.1016/j.jbc.2023.104756)
Supplement: Supporting Table S1 [file mmc2.docx]

# Supplementary Material

Table S1. Sample preparation, microscopy, data processing, and modeling.

| **Sample preparation** | |  |  |  |
| --- | --- | --- | --- | --- |
| Concentration [mg/ml] | 0.03 | | | |
| Sample volume [µl] | 5 | | | |
| Grid type | Quantifoil 100 Carbon Support Films grid: Cu 300 mesh | | | |
| Glow discharge time [s] | 25 | | | |
| Glow discharge current [mA] | 15 | | | |
| Glow discharge sample  polarity | Negative | | | |
| Glow discharge  atmosphere | residual air | | | |
| Glow discharge  pressure (Pa) | 40 | | | |
| Sample application [s] | 30 | | | |
| Sample staining with 2% uranyl acetate [s] | 45 | | | |
| Sample washing with 2% uranyl acetate [s] | immediately | | | |
| **Microscopy** | |  |  |  |
| Magnification | 92 000 | | | |
| Voltage (kV) | 200 | | | |
| Focal length (mm) | 3.4 | | | |
| Cs (mm) | 2.7 | | | |
| Objective Aperture (μm) | 100 | | | |
| Number of movies | 4946 | | | |
| Electron exposure frames /  (e–/Å^2^) | 8 frames / 30 (1 e-/frame) | | | |
| Defocus range (μm) | -0.5 to -2.0 | | | |
| Pixel size (Å) | 1.567 | | | |
| Acquisition software | TFS EPU 2 | | | |
| **Data Processing** | | | | |
| Initial model used | Ab-Initio (cryoSparc 3.2) | | | |
| Symmetry imposed | C1 | | | |
| Final particle images  (no.) | 54898 | | | |
| Map resolution (Å)    FSC threshold | 9.71  0.143 | | | |
| **Modelling** | | | | |
| Software used for model prediction | DeepMind: AlphaFold | | | |
| Software used for rigid body-fit | UCSF ChimeraX | | | |
